# Multiple-trait analyses improved the accuracy of genomic prediction and the power of genome-wide association of productivity and climate change-adaptive traits in lodgepole pine

**DOI:** 10.1186/s12864-022-08747-7

**Published:** 2022-07-23

**Authors:** Eduardo P. Cappa, Charles Chen, Jennifer G. Klutsch, Jaime Sebastian-Azcona, Blaise Ratcliffe, Xiaojing Wei, Letitia Da Ros, Aziz Ullah, Yang Liu, Andy Benowicz, Shane Sadoway, Shawn D. Mansfield, Nadir Erbilgin, Barb R. Thomas, Yousry A. El-Kassaby

**Affiliations:** 1 Instituto Nacional de Tecnología Agropecuaria (INTA), Instituto de Recursos Biológicos, Centro de Investigación en Recursos Naturales, De Los Reseros y Dr. Nicolás Repetto s/n, 1686, Hurlingham, Buenos Aires, Argentina; 2 Consejo Nacional de Investigaciones Científicas y Técnicas (CONICET), Buenos Aires, Argentina; 3 Department of Biochemistry and Molecular Biology, Oklahoma State University, Stillwater, OK 74078 USA; 4 Department of Renewable Resources, University of Alberta, 442 Earth Sciences Bldg, Edmonton, Alberta T6G 2E3 Canada; 5 Present address: Department of Forestry, New Mexico Highlands University, Las Vegas, NM 87701 USA; 6 Present address: Irrigation and Crop Ecophysiology Group, Instituto de Recursos Naturales y Agrobiología de Sevilla, Avenida Reina Mercedes, 10, 41012 Sevilla, Spain; 7 Department of Forest and Conservation Sciences, Faculty of Forestry, University of British Columbia, Vancouver, British Columbia V6T 1Z4 Canada; 8 Department of Wood Science, Faculty of Forestry, University of British Columbia, Vancouver, British Columbia V6T 1Z4 Canada; 9 Forest Stewardship and Trade Branch, Alberta Agriculture and Forestry, Edmonton, Alberta T6H 5T6 Canada; 10 Blue Ridge Lumber Inc., West Fraser Mills Ltd, Unnamed Road, Blue Ridge, Alberta T0E 0B0 Canada

**Keywords:** Quantitative genetic parameters, Genomic prediction, Genome wide association analyses, Single- and multiple-trait mixed models, Lodgepole pine

## Abstract

**Background:**

Genomic prediction (GP) and genome-wide association (GWA) analyses are currently being employed to accelerate breeding cycles and to identify alleles or genomic regions of complex traits in forest trees species. Here, 1490 interior lodgepole pine (*Pinus contorta* Dougl. ex. Loud. var. *latifolia* Engelm) trees from four open-pollinated progeny trials were genotyped with 25,099 SNPs, and phenotyped for 15 growth, wood quality, pest resistance, drought tolerance, and defense chemical (monoterpenes) traits. The main objectives of this study were to: (1) identify genetic markers associated with these traits and determine their genetic architecture, and to compare the marker detected by single- (ST) and multiple-trait (MT) GWA models; (2) evaluate and compare the accuracy and control of bias of the genomic predictions for these traits underlying different ST and MT parametric and non-parametric GP methods. GWA, ST and MT analyses were compared using a linear transformation of genomic breeding values from the respective genomic best linear unbiased prediction (GBLUP) model. GP, ST and MT parametric and non-parametric (Reproducing Kernel Hilbert Spaces, RKHS) models were compared in terms of prediction accuracy (PA) and control of bias.

**Results:**

MT-GWA analyses identified more significant associations than ST. Some SNPs showed potential pleiotropic effects. Averaging across traits, PA from the studied ST-GP models did not differ significantly from each other, with generally a slight superiority of the RKHS method. MT-GP models showed significantly higher PA (and lower bias) than the ST models, being generally the PA (bias) of the RKHS approach significantly higher (lower) than the GBLUP.

**Conclusions:**

The power of GWA and the accuracy of GP were improved when MT models were used in this lodgepole pine population. Given the number of GP and GWA models fitted and the traits assessed across four progeny trials, this work has produced the most comprehensive empirical genomic study across any lodgepole pine population to date.

**Supplementary Information:**

The online version contains supplementary material available at 10.1186/s12864-022-08747-7.

## Introduction

Interior lodgepole pine (*Pinus contorta* Dougl. ex. Loud. var. *latifolia* Engelm) is one of the most widely distributed and commercially important conifer species in Northwestern North America [1]. Traditionally, tree improvement programs focus on productivity-related traits (e.g., growth and wood quality), and rely on pedigree-based methods to characterize tested individuals [2–4]. In addition, long breeding cycles, unpredictable market demands, and climate change act individually and/or in concert to accentuate traditional tree improvement’s slow and unresponsive nature. Global climate warming since the beginning of this century, together with significant shifts in the distribution of precipitation and climate-induced insect outbreaks, has triggered a number of devastating forest mortality events worldwide [5]. For example, the most recent mountain pine beetle (*Dendroctonus ponderosae* Hopkins) outbreaks that began in 1999 had damaged over 10 million hectares of mature lodgepole pine in Alberta and British Columbia by 2007 [6]. Thus, assessing climate change-related adaptive traits such as drought tolerance and pest and disease resistance and associated secondary (defense) chemical compounds is critical to their incorporation in ongoing breeding activities for improving productivity. While productivity-related traits have been the traditional focus of quantitative genetic analyses of several tree species (including conifer species), some recent progress has been directed towards the selection of climate change-related adaptive traits [7].

Meuwissen et al. [8] introduced genomic prediction (GP) or genomic selection and the method’s potential in accelerating breeding cycles. Increasing selection intensity and improving breeding values (BVs) have been the mainstay of several animal and plant breeding programs, including forest trees [9]. The most commonly used GP method in forest tree species is the penalized ridge regression best linear unbiased predictor (RR-BLUP) method (or equivalently, genomic best linear unbiased prediction -GBLUP-; e.g., [10–12]). Several studies have compared the GP performance of different Bayesian methodologies (including BayesA, BayesB, BayesC, and BLasso) using single-trait (ST) models, where comparable results were generally found [13–19]. For example, in lodgepole pine, Ukrainetz and Mansfield [19] studied ST prediction accuracies for tree height and wood quality characteristics and found that the results from BayesC were indifferent to the GBLUP. However, non-parametric machine-learning regression methods have not been commonly applied for GP in trees species [20–23].

Unlike ST, multiple-trait (MT) GP (MT-GP) models simultaneously use the information from multiple traits to capture their correlations. Simulation studies have shown that MT-GP models can produce more accurate breeding value estimates than ST-GBLUP in animal [24] and plant [25] breeding scenarios. These benefits have also been empirically reported in a number of plants [26, 27], including tree species [12, 23, 25, 28, 29]. For instance, Cappa et al. (2018) demonstrated a 2 to 4% increase in the theoretical accuracy of a low heritability trait (tree height, HT, ${\hat{h}}^2$ = 0.15) using MT-GBLUP model, when leveraging the genetic correlation with diameter at breast height (DBH, ${\hat{h}}^2$ = 0.32). Additionally, in a 10-fold cross-validation analysis of a *Pinus taeda* L. population, Jia and Jannink [25] observed better prediction accuracy (0.48 vs. 0.30) for fusiform rust (*Cronartium quercuum* Berk. Miyable ex Shirai f. sp. *fusiforme*) assessed as gall volume with MT-BayesCπ model than from an ST model.

In addition to GP, the whole genome approach allows for the investigation of traits’ genetic architecture (defined as the number of genes affecting a quantitative trait), allelic effects on phenotypes, and the frequency distribution spectrum of alleles at these genes [30]. In MT genome-wide association (GWA) studies (MT-GWA), it is known that correlations between traits reduce false positives and increase the statistical power of association tests [31–33]. Compared to ST-GWA, and from a biological perspective, MT-GWA enhances pleiotropy interpretation [32], when a specific locus affects multiple traits [34]. In a GWA human study, Watanabe et al. [35] compiled a catalog of 4155 GWA results across 2965 unique traits from 295 studies, and found that 90% of the genes were associated with more than one trait, highlighting that pleiotropy plays an important role in the genetic architecture of complex traits. Over the past two decades, ST-GWA studies that fit one trait at a time have established the genetic architectural foundations for a number of growth and wood quality phenotypes in forest tree species [36–40]. Except for a small number of physiological traits [33, 38–40, Liu et al. submitted] little is known about the detailed genetic basis and the predictability of adaptive attributes of coniferous species, which are key to global biogeochemical cycles and climate regulation [41, 42].

Here, we assessed 1490 trees from a lodgepole pine population being tested in four open-pollinated progeny trials in central Alberta, where we characterized not only growth and wood quality traits, but also phenotypes related to pests and drought resistance and defense chemical (monoterpenes) traits with 25,099 SNP markers. The main objectives of this study were to (1) identify genetic markers associated with these productivity- and adaptability-related traits and determine their genetic architecture, and to compare the marker detected by ST- and MT-GWA GBLUP models; (2) evaluate and compare the accuracy and control of bias of the genomic predictions for these traits underlying different ST and MT parametric (BayesC, Blasso, BRR, and GBLUP) and non-parametric (Reproducing Kernel Hilbert Spaces, RKHS) GP methods. Additionally, we estimated the quantitative genetic parameters (including heritability and genetic correlations) within- and across-sites of these productivity- and adaptability-related traits.

## Results

### Genomic heritability estimates and correlations between traits within- and across-sites

Overall, narrow-sense heritability estimates based on genomic data across the 15 studied traits ranged from 0.01 to 0.80 with an average of 0.42 (Table 1). Substantial cross-site heritability estimates were observed for growth (DBH and HT) and wood quality traits (wood density, WD; and microfibril angle, MFA), being significantly lower for SWAN (DBH and WD), TIME (DBH) and VIR (WD and MFA). Pest resistance traits (western gall rust, WGR; and mountain pine beetle, MPB) also had low-to-moderate heritability estimates (range: 0.40–0.68 and 0.01–0.65 for WGR and MPB, respectively). The dendrochronological growth decline index (DECL) produced low-to-moderate heritability estimates for three out of four sites (range: 0.24–0.50), but near-zero heritability was obtained for the VIRG site (0.01). While the stable carbon isotope ratio (δ^13^C) values showed consistently moderate-to-high heritability estimates across sites (range: 0.42–0.64), monoterpene compounds produced variable estimates, with values ranging between 0.20 and 0.80. Finally, total monoterpenes showed slightly lower heritability estimates than individual monoterpene compounds (range: 0.25–0.33).

**Table 1:** Narrow-sense heritability estimates and their approximate standard error (SE), for each of the growth, wood quality, pest resistance, drought tolerance and chemical defense assessed traits at four progeny test sites in a lodgepole pine population. Heritabilies were estimated using the genomic- relationship matrix (***G***-matrix) constructed using 25 K SNPs. See text for site and trait abbreviations

Trait / Site^***b***^	JUDY	VIRG	SWAN	TIME
**HT**	0.767 (0.201)	0.503 (0.221)	0.616 (0.153)	0.432 (0.180)
**DBH**	0.495 (0.178)	0.464 (0.207)	0.186 (0.136)	0.091 (0.137)
**WGR**	0.465 (0.174)	0.399 (0.213)	0.648 (0.165)	0.678 (0.197)
**WD**	0.617 (0.213)	0.293 (0.216)	0.203 (0.163)	0.576 (0.190)
**MFA**	0.320 (0.171)	0.064 (0.184)	0.452 (0.153)	0.219 (0.146)
**δ**^**13**^**C**	0.537 (0.198)	0.423 (0.216)	0.638 (0.178)	0.434 (0.167)
**DECL**^***a***^	0.497 (0.222)	0.005 (0.190)	0.239 (0.173)	0.400 (0.206)
**MPB**	0.140 (0.163)	0.651 (0.211)	0.353 (0.141)	0.013 (0.008)
**α-pinene**^***a***^	0.681 (0.189)	0.355 (0.205)	0.317 (0.168)	0.503 (0.176)
**β-pinene**^***a***^	0.298 (0.161)	0.491 (0.211)	0.686 (0.142)	0.547 (0.174)
**myrcene**^***a***^	0.376 (0.196)	0.281 (0.196)	0.237 (0.146)	0.374 (0.175)
**limonene**^***a***^	0.523 (0.207)	0.613 (0.228)	0.495 (0.161)	0.200 (0.148)
**β-phellandrene**^***a***^	0.437 (0.184)	0.671 (0.206)	0.795 (0.149)	0.661 (0.171)
**terpinolene**^***a***^	0.233 (0.179)	0.566 (0.201)	0.373 (0.149)	0.547 (0.175)
**total monoterpene**^***a***^	0.266 (0.183)	0.326 (0.194)	0.256 (0.151)	0.247 (0.167)

^*a*^ Logarithmic transformed

**Fig. 1:**
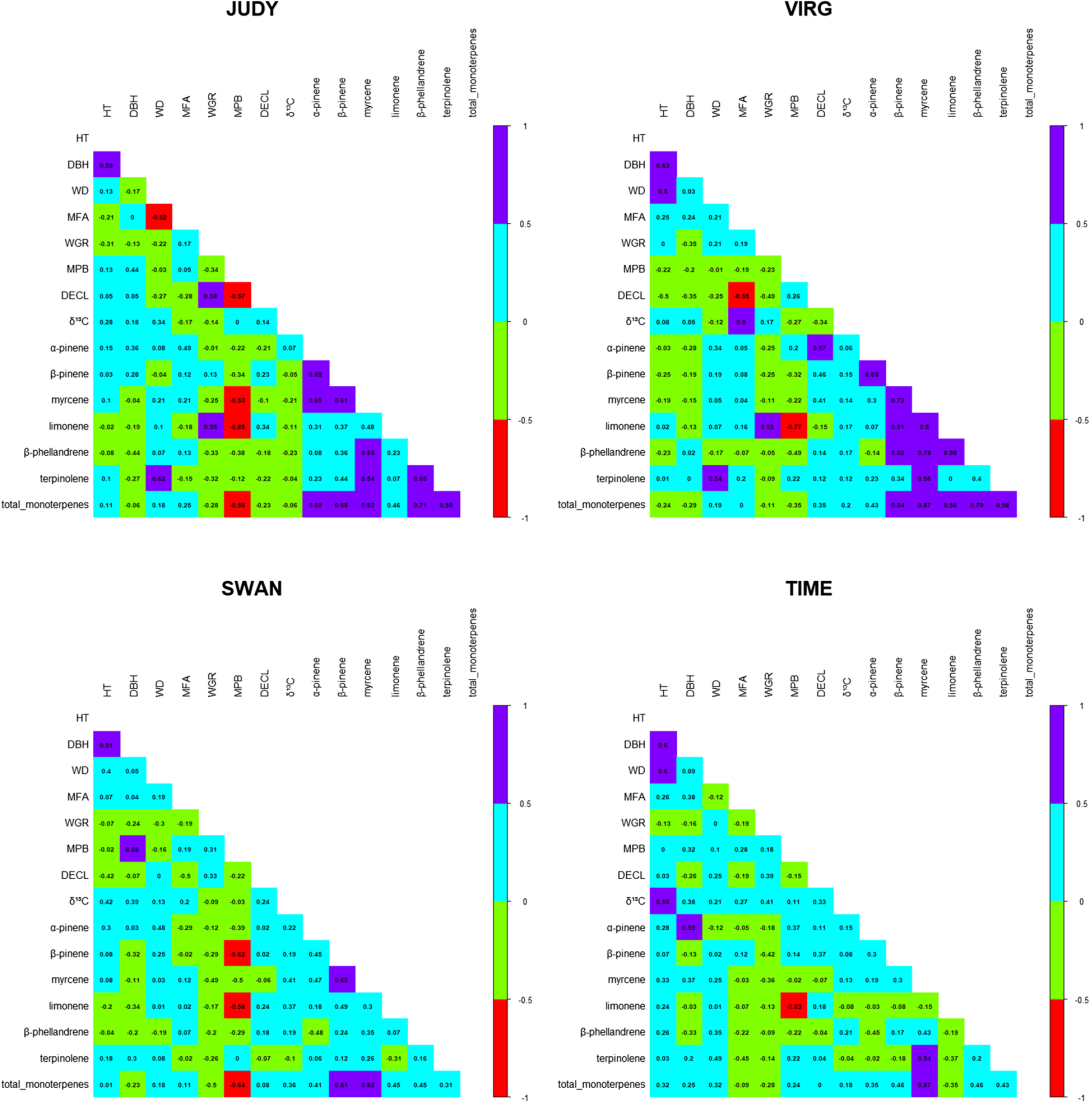
Genomic-based multiple-trait estimates of genetic correlation among the 15 traits studied. Colours reflect the genetic correlation strength, with red and green indicating negative correlations and light blue and violet reflecting positive correlations in lodgepole pine, respectively. See text for site and trait abbreviations

Overall, genetic correlations between traits ranged from − 0.85 to 0.92 (Fig. 1 and Supplementary Table S1). Growth traits showed low and negative genetic correlations with WGR (range: − 0.35 - 0.00) and low-to-moderate correlations with MPB (depending on the sites, either positive (JUDY, SWAN, and TIME) or negative (VIRG); range: − 0.22 - 0.58). The correlations between growth traits and DECL were also low-to-moderate and generally negative for VIRG, SWAN and TIME (range: 0.03 - 0.50), and low and positive but not statistically significant (with a relatively large standard error) for JUDY (0.05). The correlation between growth traits and δ^13^C varied from 0.05 to 0.55 with lower values for JUDY and VIRG and higher values for SWAN and TIME.

With the exception of WD at TIME (0.20), wood quality traits were generally negatively correlated with DECL (range: 0.00 - 0.27 for WD and − 0.19 - 0.55 for MFA). Meanwhile, WD was low and positively or negatively correlated with δ^13^C values (range: − 0.12 - 0.34). Except for JUDY (− 0.17), MFA generally produced low-to-high positive correlations (range: 0.14 - 0.80) with δ^13^C.

Low-to-moderate and negative genetic correlations between the two pest resistance traits (WGR and MPB) were detected at JUDY (− 0.23) and VIRG (− 0.34) but positive at SWAN (0.31) and TIME (0.18). In general, with the exception of WGR and limonene at JUDY (0.55) and VIRG (0.53), negative genetic correlations between WGR and monoterpene compounds (including total monoterpenes) were observed across all sites. Similarly, MPB showed a negative correlation with monoterpene compounds and total monoterpenes, and as expected, these correlations were stronger with limonene (range: − 0.58 - 0.85) followed by β-phellandrene, the most abundant monoterpene compounds in lodgepole pine phloem (range: − 0.22 - 0.49).

Except for VIRG (− 0.34), DECL showed low and positive genetic correlations with δ^13^C values across sites (range: 0.14–0.33). Low-to-moderate and generally positive correlations were found between DECL and monoterpene compounds, including the total monoterpenes. Correlation estimates between δ^13^C values and monoterpene compounds and total monoterpenes also varied across sites, with generally low and negative values for JUDY (range: − 0.10 - 0.23), except with limonene (0.34) and β-pinene (0.23), and generally positive for the remaining sites (range: − 0.15 - 0.57).

Finally, with some variation across sites (e.g., average 0.49 for JUDY and 0.14 for TIME), the genetic correlations between monoterpene compounds (including total monoterpenes) were generally moderate-to-high and positive. A few exceptions of negative correlations can be seen between α-pinene and β-phellandrene.

**Fig. 2:**
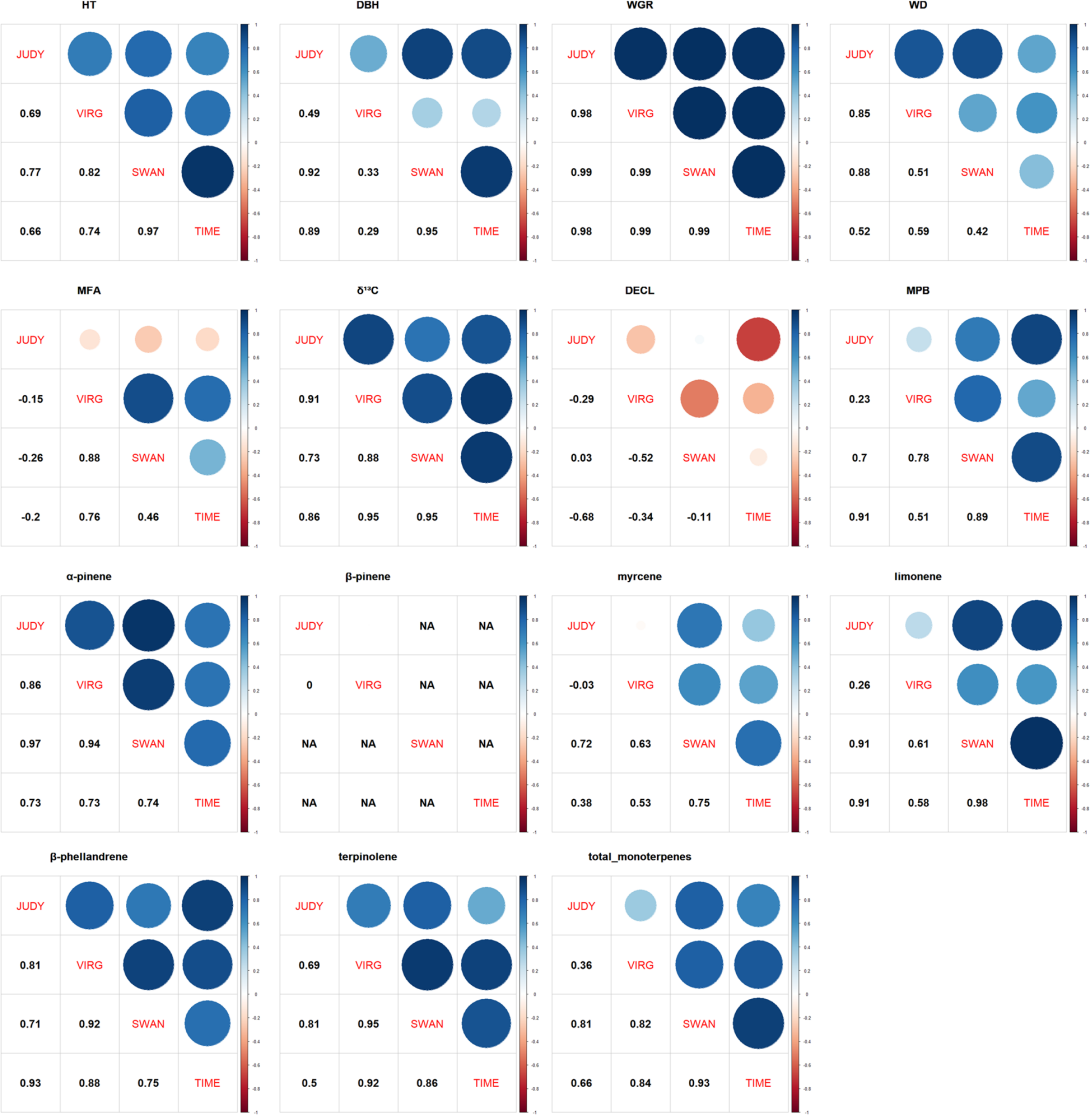
Genomic-based multiple-site estimates of genetic correlations between the four lodgepole pine progeny test sites. Genetic correlation estimates are shown in each cell below the diagonal, with colour and size of circle reflecting the genetic correlation strength. The red and blue circles reflect negative and positive correlations, respectively, and small (weaker) and larger (stronger) circles indicate the strength of the correlation, shown above diagonal. See text for trait abbreviations

In general, we observed high and positive genetic correlations between sites (i.e., low genotype by environment interactions, G × E) (50 out of 72 pairs were > 0.7 and 60 were > 0.4) and with relatively small standard errors (Fig. 2 and Supplementary Table S2). Inconsistent genetic correlations and larger standard errors were observed for MFA and DECL, with generally low and relatively large standard errors. Due to these inconsistencies, these two traits were not included in the multiple-trait GP and GWA analyses. The lowest genetic correlations among pairs of sites were between JUDY and VIRG (average across traits 0.48) and JUDY and TIME (average across trait 0.58), while the average for the rest of the pairs were strong (higher than 0.64). We suspected that VIRG’s low genetic correlations with other sites were likely caused by the fire that occurred on this site in May 1998 (see Discussion below).

### Genome-wide association (GWA)

**Fig. 3:**
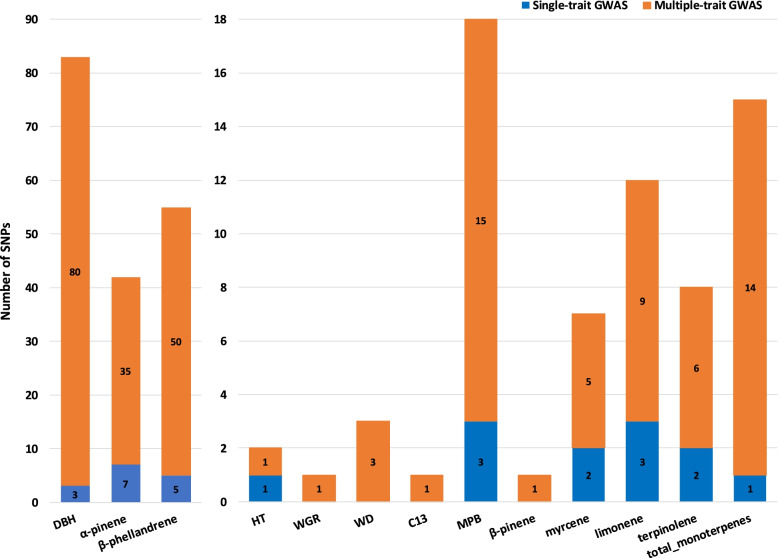
Number of total significant (*p*-values < 1.99 × 10^− 06^) SNPs identified by the single-trait (blue) and multiple-trait (orange) GWA analyses in lodgepole pine. A total of 17 SNPs was identified for both GWA analyses for the traits HT (1), MPB (2), α-pinene (7), limonene (2), and β-phellandrene (5). See text for site and trait abbreviations

Manhattan plot generally shows similar profiles between ST- and MT-GWA analyses for the 13 traits studied (Supplementary Fig. S1). The ST- and MT-GWA analyses with 25,099 SNPs for each of the 13 studied traits identified a total of 27 and 221 SNPs, respectively, and all these SNPs passed the Bonferroni correction *p*-value cutoff of 1.99 × 10^− 06^ (−logP = 5.7) (Fig. 3). Therefore, we observed a total of 248 significant SNPs for both ST- or MT-GWA; however, 17 SNPs overlapped between ST and MT results (HT (1), MPB (2), α-pinene (7), limonene (2), and β-phellandrene (5)). Overall, the MT-GWA analysis appeared to be more effective in identifying 204 unique SNPs that were not detected by the ST-GWA analysis. In contrast, only 10 SNPs were found to be unique in the ST-GWA. In addition, the greatest number of significant SNPs identified with both GWA models (ST and MT) were associated with DBH (83), β-phellandrene (50) and α-pinene (35), while the lowest number of identified SNPs were associated with HT, WGR, δ^13^C, and β-pinene with only one SNP overlapping.

The MT-GWA analysis identified 28 SNPs that simultaneously contributed to multiple traits. These include associations between growth traits (DBH and HT (1 SNP)), growth and defense monoterpenes (DBH and α-pinene (2 SNPs) and DBH and β-phellandrene (1 SNP)), wood quality and defense monoterpenes (WD and β-phellandrene (1 SNP) and WD and total monoterpenes (1 SNP)), pest resistance and defense monoterpenes (MPB and limonene (4 SNPs)), and defense monoterpenes (α-pinene and β-phellandrene (14 SNPs), and finally myrcene and total monoterpenes (4 SNPs)). However, the ST-GWA analysis only identified five SNPs with potential pleiotropic effects (details in Table 2).

**Table 2:** Number of significantly associated SNPs with a Bonferroni correction *p*-value cutoff of 1.99 × 10^−06^ for single-trait and multiple-trait GWA analyses (diagonal), and number of common significant SNP markers across different traits by single-trait (above diagonal) and multiple-trait (below diagonal) GWA analyses in lodgepole pine. See text for site and trait abbreviations

	HT	DBH	WGR	WD	δ^**13**^C	MPB	α-pinene	β-pinene	myrcene	limonene	β-phellandrene	terpinolene	total_monoterpenes
**HT**	1	–	–	–	–	–	–	–	–	–	–	–	–
**DBH**	1	83	–	–	–	–	–	–	–	–	–	1	–
**WGR**	–	–	1	–	–	–	–	–	–	–	–	–	–
**WD**	–	–	–	3	–	–	–	–	–	–	–	–	–
**δ**^**13**^**C**	–	–	–	–	1	–	–	–	–	–	–	–	–
**MPB**	–	–	–	–	–	16	–	–	–	2	–	–	–
**α-pinene**	–	2	–	–	–	–	35	–	–	–	–	–	–
**β-pinene**	–	–	–	–	–	–	–	1	–	–	–	–	–
**myrcene**	–	–	–	–	–	–	–	–	7	1	–	–	1
**limonene**	–	–	–	–	–	4	–	–	–	10	–	–	1
**β-phellandrene**	–	1	–	1	–	–	14	–	–	–	50	–	–
**terpinolene**	–	–	–	–	–	–	–	–	–	–	–	8	–
**total_monoterpenes**	–	–	–	1	–	–	–	–	4	–	–	–	15

**Fig. 4:**
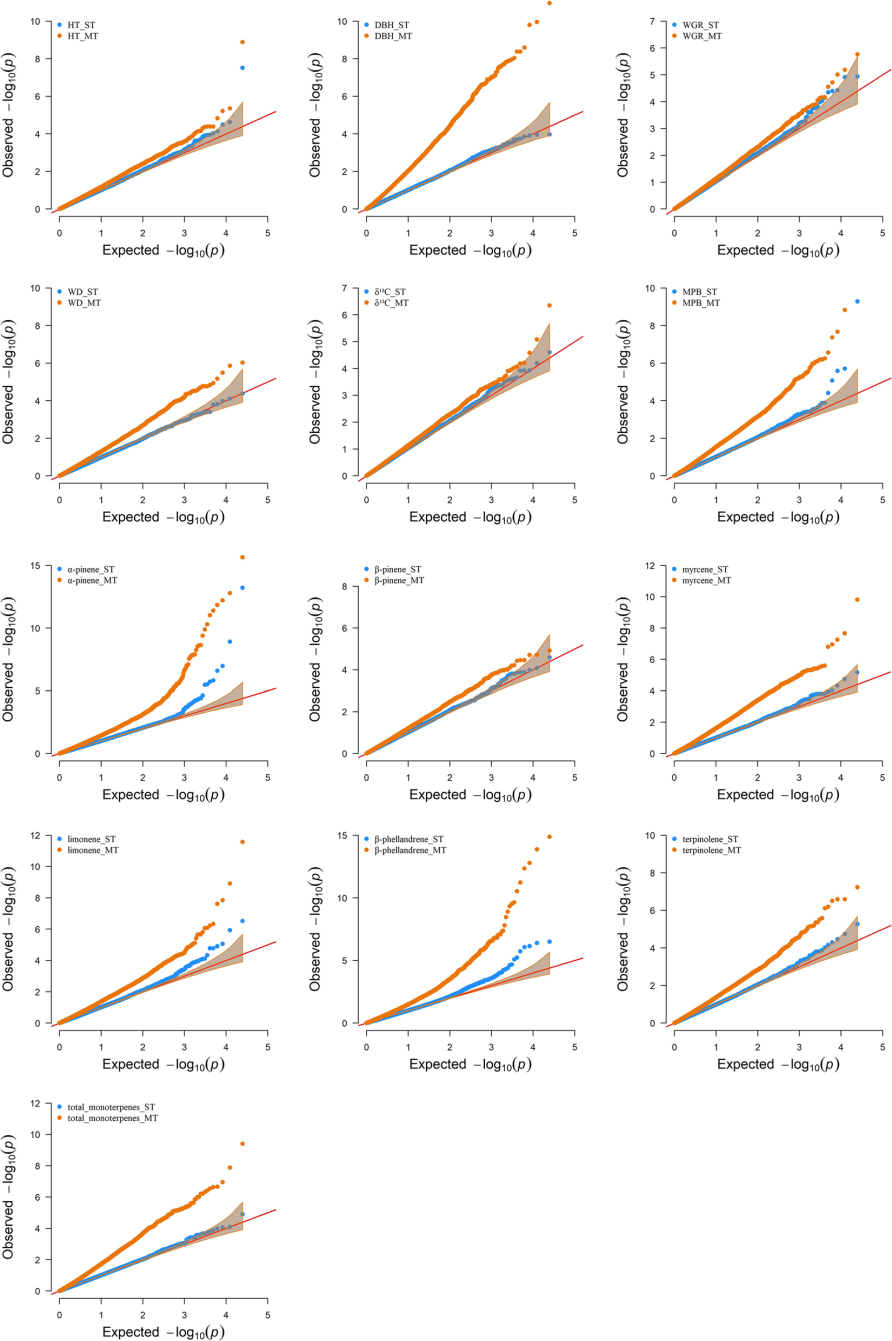
Quantile-quantile (Q-Q) plots for genome-wide association (GWA) analyses based in single-trait (ST, blue) and multi-trait (MT, orange) models for 13 traits studied in lodgepole pine. Q-Q plot is used to assess the number and magnitude of observed associations between genotyped SNPs and traits under study, compared to the association statistics expected under the null hypothesis of no association. See text for trait abbreviations

**Fig. 5:**
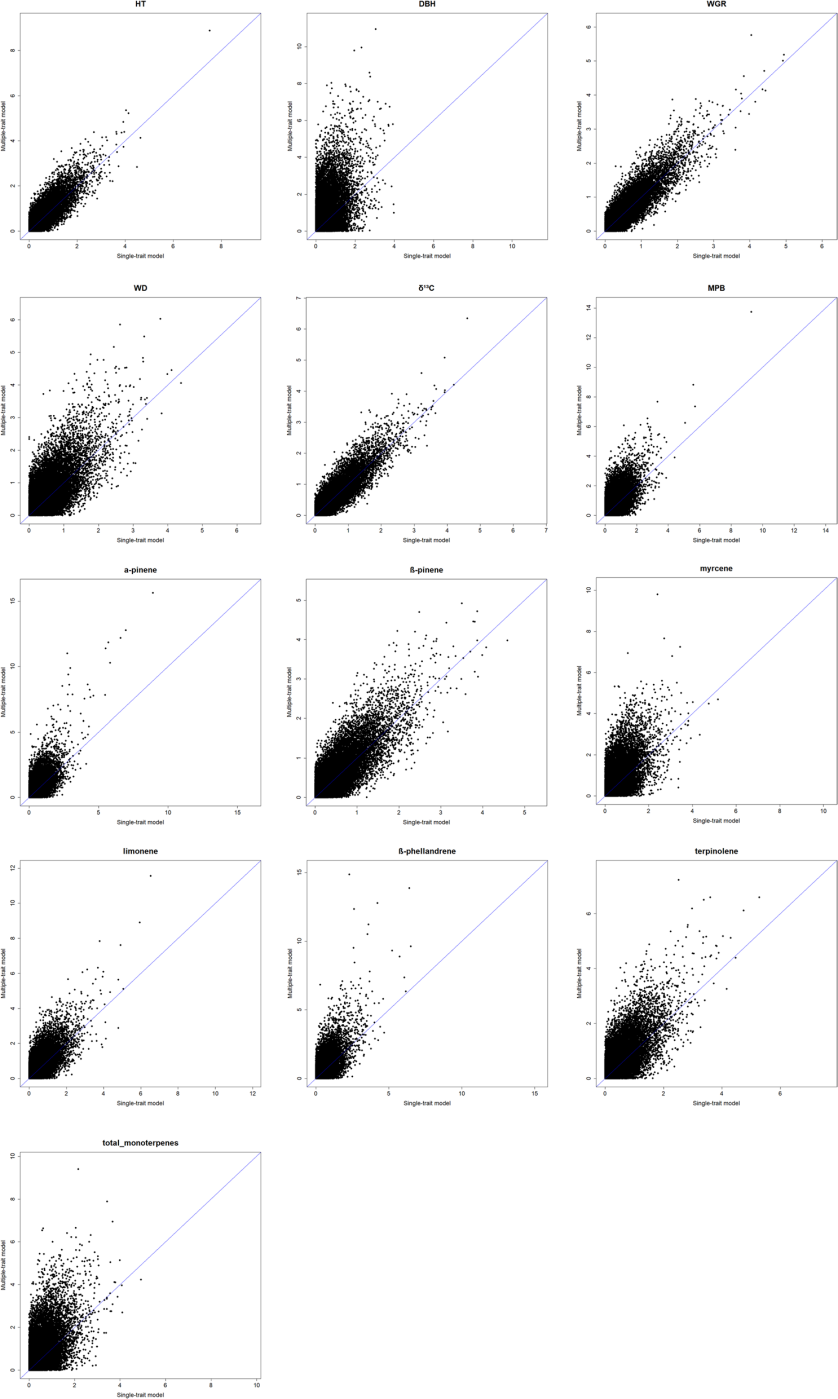
Scatterplots of *p*-values in -log_10_(*p*-value) scale for the single-trait (*x*-axis) and multiple-trait (*y*-axis) GWA analyses in lodgepole pine for 13 traits studied. Note as several points (markers) are positioned above the blue line (i.e., deviated from the diagonal (regression line y = x), suggesting the multiple-trait association analysis increased the power as compared to the single-trait analysis. See text for trait abbreviations

QQ plots showed a clear improvement for MT associations compared to their corresponding ST analyses (Fig. 4). The scatter plots, where the –log_10_(*p*-value) for each trait obtained from ST-GWA and MT-GWA were compared, also suggest an increase in the power of MT association analysis (Fig. 5). Further evidence of the strength of the MT-GWAS can be seen in the number of the SNPs deviated from the diagonal (regression line y = x in Fig. 5).

### Genomic prediction (GP)

After performing the Tukey’s test (α = 0.05), it was found that the average prediction accuracy across the studied traits of the five ST-GP models (BayesC, BLasso, BRR, GBLUP, and RKHS) did not differ significantly from each other (range: 0.410–0.435), with the highest values for the non-parametric RKHS-GP model in 11 out of the 13 studied traits (Supplementary Table S3). Similarly, the ST-RKHS approach showed the lowest prediction bias among all five ST-GP models studied (average across traits = 0.851), statistically significantly different from the other four ST-GP models.

**Fig. 6:**
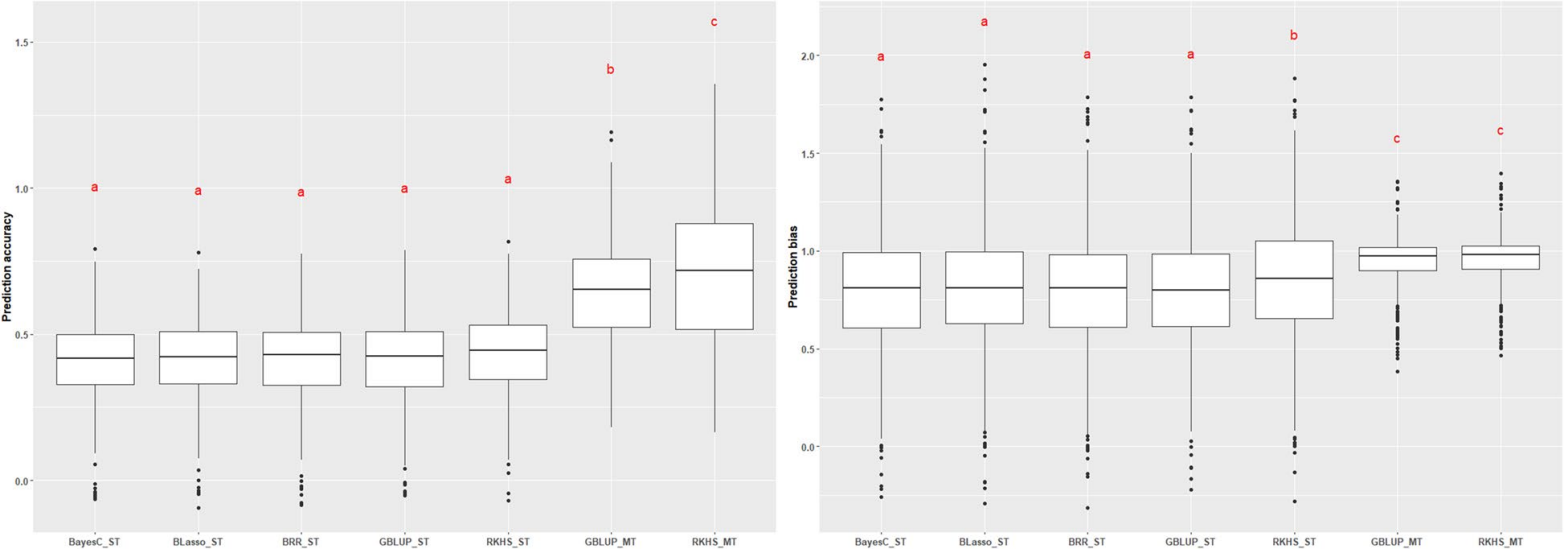
Average prediction accuracy and prediction bias across 13 traits using different genomic selection methods for five single- (ST) and two multiple-trait (MT) models in lodgepole pine. Common letters above box-plots are not significantly different (α = 0.05) according to the Tukey test. See text for ST and MT model abbreviations

For MT-GP models, GBLUP and RKHS demonstrated significantly higher average prediction accuracies across traits than the five ST models. Additionally, within the MT-GP approaches, the MT-RKHS model provided significantly higher predictability than the MT-GBLUP (average 0.703 for MT-RKHS vs. 0.644 for MT-GBLUP, 9.2%). Though not statistically significant, the averaged prediction bias across traits of the MT-GBLUP and MT-RKHS GP models were found to be lower than those of the ST-GP models (average across traits of 0.948 vs. 0.959, respectively; Fig. 6 and Supplementary Table S3).

**Fig. 7:**
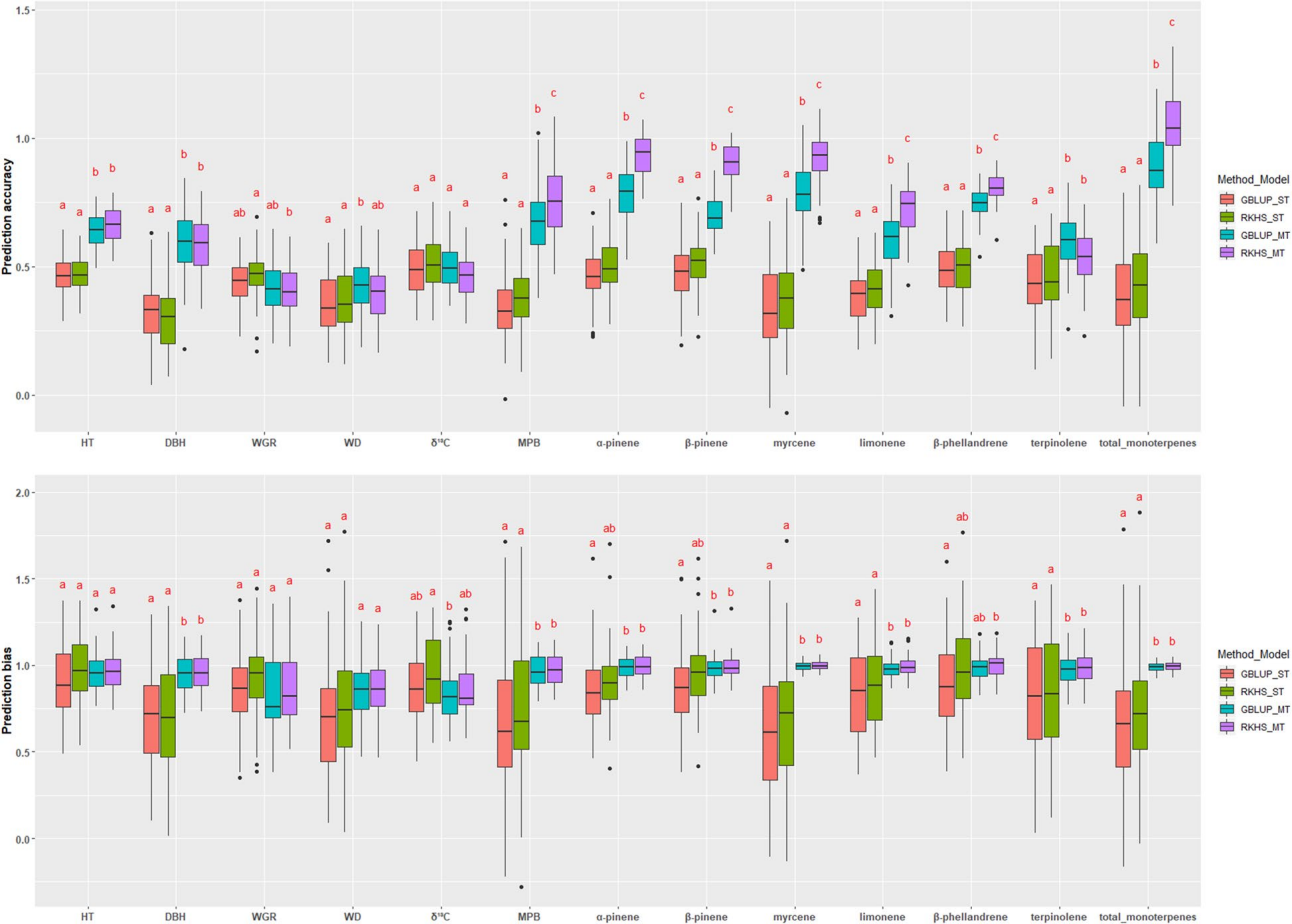
Average prediction accuracy and prediction bias using different genomic selection methods for two single-trait (ST) and two multiple-trait (MT) models for 13 traits studied in lodgepole pine. Within each trait, common letters above box-plots are not significantly different (α = 0.05) according to the Tukey test. See text for model and trait abbreviations

Regarding each of the studied traits (i.e., within trait), the MT-GBLUP- and RKHS-GP models showed higher prediction accuracy and lower bias (most were statistically significant, *p*-value < 0.05) than those of their respective ST-GP models, with the exception to the prediction accuracy of WGR and δ^13^C traits (Fig. 7 and Supplementary Table S3). However, prediction performance (increment in the prediction accuracy or reduction in the bias) varied significantly among the studied traits. For example, comparing GBLUP and RKHS for the ST-GP models, ST-RKHS showed higher prediction accuracy and lower bias than the ST-GBLUP for all traits (but not statistically significant, *p*-value > 0.05), except for the prediction accuracy of DBH. However, MT-RKHS outperformed the MT-GBLUP model, in terms of accuracy, in eight out of the 13 traits studied, while lower bias was calculated for all traits in the MT-RKHS compared to those values predicted by MT-GBLUP (Supplementary Table S3).

## Discussion

While assessing the prediction accuracy based on genomic information and the dissection of the genetic architecture of productivity-related traits has been a common goal in recent forest tree species research, less effort has been directed towards climate-adaptive traits. In this study, 1490 lodgepole pine trees from four open-pollinated progeny trials were genotyped with 25,099 SNPs and phenotyped for 15 growth, wood quality, pest resistance, drought tolerance, and defense chemical (monoterpenes) traits. Several parametric and non-parametric and single- (ST) and multiple-trait (MT) genomic prediction (GP) and genome-wide association (GWA) models were evaluated and compared. The main results showed that, firstly, in the GWA analysis, the MT model showed a clear improvement and identified more significant SNPs than the ST approach. Furthermore, some of these SNPs simultaneously were associated with multiple traits. Secondly, in the GP analysis, while similar prediction efficiencies and bias were observed regardless of the ST-GP model used, the MT-GP models (GBLUP and RKHS) increased the prediction accuracy and reduced the prediction bias considerably as compared to those of the ST models. In general, the non-parametric RKHS model showed higher prediction accuracy, and lower bias for both ST and MT models across the 13 traits studied.

### Genome-wide association (GWA)

Single- (ST) and multiple-trait (MT) GWA analyses using a linear transformation of the genomic breeding values from their respective GBLUP models were carried out to identify significant SNPs associated with the studied 15 traits. We used the MT-GBLUP models’ genomic breeding values to increase the power of the association tests and detect associations with potential pleiotropic effects.

Dissecting the genetic architecture of complex traits is essential to understanding quantitative trait variation and evolvability [36]. However, due to the lack of genomic knowledge, such as reference assemblies and functional annotations, genetic association studies in conifer have primarily been carried out using candidate-genes or focused on specific genomic regions [43, 44]. Recently, genome-wide genotyping approaches have been applied to several conifer species, including *Pinus taeda* [36, 45] and *Picea abies* (L.) Karst [40]. In lodgepole pine, until now, only one other association study that identified 11 SNP loci with an adaptive phenotype, serotiny, was available using 98 trees genotyped with more than 95,000 SNPs [46]. Our results demonstrate that small differences exist in the architecture of the different productivity and climate resilience traits studied, with no evidence of alleles of large effect (Supplementary Fig. S1). Thus, these traits’ architecture is complex following the infinitesimal model. Here we have uncovered a quantitative aspect- with a total of 231 trait-associated SNPs for 13 traits identified (Table 2). Armed with the increased statistical power of the MT-GWA models, recently, there is a growing appreciation that many genetic variants can influence multiple traits simultaneously [47]. For instance, Chhetri et al. [48] found four SNPs with suggestive associations using ST-GWA for 14 morphological and physiological traits in *Populus trichocarpa*, while using MT-GWA for 12 combinations of a subset of these 14 traits, 32 SNPs passed the suggestive association *p*-value cutoff. Similar cases in other species such as dairy cattle [49] and fish [50] have also been observed. Based on our study, we suspect that the increase in statistical power could be the result of the genetic correlations between these traits (Fig. 1 and Supplementary Table S1).

In our ST-GWA analysis, 17 out of 27 associations were confirmed by the MT-GWA analysis: 7 for α-pinene, 5 for β-phellandrene, 1 for HT, 2 for MPB, and 2 for limonene. However, ten associations were only seen in the ST-GWA results: 3 for DBH, 2 for myrcene, 2 for terpinolene, 1 for total monoterpenes, 1 for MPB, and 1 for limonene. Given that the MT-GWA analysis failed to confirm these ten associations, and as indicated [50], we may assume that these trait associations identified by the ST-GWA analysis are spurious.

Furthermore, our results suggested that MT-GWA analyses may potentially capture a higher number of pleiotropic effects than those from ST-GWA analysis (28 vs. 5, respectively) (Table 2). However, Fernandes et al. [51], using publicly available molecular marker data from *Zea mays* L. (maize) and *Glycine max* L. (soybean), suggested that the MT-GWA model tended to yield high spurious pleiotropy detection rates. They recommended that future studies should use a combination of ST- and MT-GWA models for distinguishing between true and spurious pleiotropy.

Additive GBLUP models have been considered in the ST- and MT-GWA analyses performed in this study, and dominance has been omitted mainly for reasons of computational efficiency. Some presence of genetic dominance effects of complex growth and adaptive traits has been previously reported in GWA conifers studies in clonally replicated trees of *Pinus taeda* L. [44, 52], and in *Pseudotsuga menziesii var. menziesii* [53]. However, when the genetic architecture of a trait is in fact mostly additive, ignoring the dominance effect provides inferences that are nonetheless adequate [54]. In our study, the additive single-trait GBLUP model gave slightly small values of Akaike information criterion (AIC) (i.e., better fits) than the additive and dominance GBLUP model for all the productivity and climate-adaptability complex traits studied, with only one exception β-phellandrene (results not shown). Moreover, the dominance variance in nine out of 13 traits was less than 14% of the total genetic variance (in the remaining three traits it did not exceed 20%), and the ratios of dominance to additive variances did not exceed 0.256. These results confirm that the genetic architecture of the studied traits is mostly additive, and the ST- and MT-GBLUP that model only additive effects have accurately characterized the genetic architecture of these phenotypes.

### Genomic prediction (GP)

In all 13 traits examined for genomic prediction, except for the slightly higher accuracy and lower bias observed in the RKHS model, all other models yielded comparable results (Supplementary Table S3). In terms of prediction accuracy, Daetwyler et al. [55] demonstrated that the genetic architecture of traits and properties of the genomes are the major factors contributing to the discrepancy between parametric and non-parametric prediction models. Our results suggest that no alleles of large effect existed, or they are at very low frequency (Supplementary Fig. S1). Reviews of both plant and animal literature have generally concluded that the Bayesian methods outperform the RR-BLUP (or equivalently GBLUP) for traits under the oligogenic model (i.e., trait controlled by few markers of moderate to large effect), whereas for polygenic traits, the GBLUP is expected to perform better. In forest tree species, several studies were conducted to compare the predictive performance of GBLUP and Bayesian methods. However, non-parametric models were rarely used for GP in forest trees [21, 22]. All of these studies showed that the GBLUP approach has prediction accuracies comparable to the Bayesian alphabets, as well as to non-parametric methods, which suggests that growth and wood quality productivity traits in eucalypts [13, 16, 17, 21], pines [10, 15, 18, 56], and spruce [12, 14, 22, 57], are complex and adequately fit into the assumption of the infinitesimal model [58]. However, when examining traits known to have an oligogenic basis, Resende et al. [18] found that BayesA and BayesCπ were superior to the ridge regression-BLUP for fusiform rust disease phenotype in loblolly pine.

When penalized and Bayesian GP models are compared with the non-parametric RKHS approach, in *P. abies*, GBLUP, BLasso, and RKHS had similar accuracies for three wood quality traits, but a slightly higher accuracy was observed for BRR for tree height [22]. However, in *Eucalyptus*, in agreement with our results, RKHS yielded a slightly better prediction accuracy for six out of eight growth and wood quality traits studied than the other three GP models (GBLUP, ridge regression-BLUP, and BLasso) [21].

Future forestry genetic improvement requires recurrent selection of all desirable traits, such as growth, wood quality, pest resistance, drought tolerance, and defense chemical (monoterpenes) traits. In our current study, the advantage of MT-GP over ST-GP models is demonstrated with the improved accuracies and reduced bias (Figs. 6 and 7 and Supplementary Table S3). These results are in line with previous studies in forest trees where up to three traits were used in the MT models [25, 28]. However, also shown in our results, the advantage of MT-GP models can be trait-dependent and sometimes, negligible [12, 23, 29]. Genetic correlation between traits is the basis for the benefit of MT-GP models [25]. Consequently, the MT-GP models´ efficiency, relative to ST-GP, depends on the genetic correlations between traits. Using simulation, Calus and Veerkamp [59] reported that the accuracy increase was higher when the genetic correlation was greater than 0.5.

In addition, using MT-GP models, the predictability of low-heritability traits can benefit from leveraging the genetic correlation with high-heritability traits [25]. For example, myrcene, a trait with relatively low heritability (0.32, Table 1) but in sizable genetic correlation with the other traits (average across traits 0.55, Supplementary Table S1), MT-GP showed significant improvement with a 133% increase in prediction accuracy (see ST-GLUP vs. MT-GBLUP in Supplementary Table S3). On the other hand, δ^13^C, a trait with a relatively high heritability (0.51, Table 1) but low correlation with the other traits (average across traits 0.08, Supplementary Table S1), the benefit of MT-GP was, as a result, not significant, showing the lowest increment of 3.0% gain in prediction accuracy (Supplementary Table S3).

Finally, in our MT-GP analyses, non-parametric RKHS generally outperformed GBLUP in predictive ability and bias. RKHS, using a non-linear Gaussian kernel, is expected to be more effective than the linear kernel (GBLUP) for capturing the underlying genome complexity, including cryptic interactions between markers [60], non-additive genetic effects like dominance and epistatic interactions [61], environmental interactions [62, 63], as well as other interactions that are not considered in standard quantitative genetic models [64]. Our empirical GWA analyses revealed the complex genetic architecture of the studied traits (Supplementary Fig. S1), and this can explain the higher efficiency of the RKHS model in capturing small and complex interactions not considered in the linear GP models. Similar findings in cereal crops have confirmed the advantage of using non-linear kernels to increase predictability [60, 62, 65–67]. Overall, given the number of GP models fitted and the traits assessed across four progeny trials, this study has shown the most comprehensive empirical GP analysis in a lodgepole pine population to date.

## Conclusion

We used dense genotypic information to perform a GP and GWA analyses on 15 growth, wood quality, pest resistance, drought tolerance, and defense chemical (monoterpenes) traits assessed in four lodgepole pine progeny trials. This study showed that MT-GWA analyses provided a substantial improvement in the number of significant SNP markers identified compared to ST-GWA and the potential of identifying pleiotropic effects of individual genes, confirming the increase in statistical power of the MT-GWA models. In addition, we found several SNP markers (231) significantly associated with productivity- and adaptability- traits. For genomic prediction, the five different parametric and non-parametric ST-GP methods produced very similar predictive ability and bias. However, slightly higher prediction accuracy and lower bias were generally observed for the non-parametric RKHS GP model. The model comparison showed that MT-GP models yield a relatively higher prediction accuracy and lower bias than the ST models. We also demonstrated the superiority of the MT RKHS approach to its linear counterpart, GBLUP. Furthermore, the MT-GP and GWA models used in this study do not consider the causal relationships between traits [68]. A future direction may be to use structural equation models (SEM) theory further to investigate the functional relationships between these complex productivity- and adaptability-related traits, with the objective of enhancing the prediction accuracy of genomic BVs, and facilitating a better understanding of the genotype-phenotype associations in lodgepole pine.

## Methods

### Genetic material and trial description

Four open-pollinated (OP) lodgepole pine progeny tests (Judy Creek: JUDY, Virginia Hills: VIRG, Swan Hills: SWAN, and Timeau: TIME) in the Region C breeding program [69] owned and managed by West Fraser - Blue Ridge Lumber, Inc. were used in this study (Supplementary Fig. S2). This breeding population contains 224 OP families selected from five natural stands (i.e., provenances; Deer Mountain, Inverness River, Judy Creek, Swan Hills, and Virginia Hills) within the Region C [70]. The field experimental design was the same at all progeny tests: a “set in reps” design with five replications (blocks), with 21 sets per replication, and trees within sets were planted in 4-tree row plots at a 2.5 × 2.5 m spacing [71]. Further details about these test sites can be found in Cappa et al. [72] and in Supplementary Table S4.

### Traits evaluated

Two growth traits, diameter at breast height (1.3 m; DBH) and tree height (HT), were measured at age-30. Wood density (WD) from 5 mm increment cores was measured at approximately breast height. See details on core sampling, transportation and analyses in Cappa et al. [7]. Wood density data represents the relative density on an oven-dry weight basis. Average WD was calculated as the weighted WD of individual tree rings weighted by their ring width, to better represent the density of the whole tree [7]. Microfibril angle (MFA) was determined by X-ray diffraction on the radial face of the individual growth rings (see Ukrainetz et al. [73] for details).

Based on Cappa et al. [72], *Endocronartium harknessii* (J.P. Moore) Y. Hirats (western gall rust, WGR) infection severity was assessed at age 36 using a scoring system with seven ordered discrete categories ranging from no gall to deceased (i.e., lowest rating having no galls to highest rating having a high number of galls) [74]. Very few trees were assessed in categories 3, 5, and 7 across test sites, therefore these categories were merged with the original 2, 4, and 6 categories, respectively, resulting in four-category resistance ratings, as indicated by Cappa et al. [72]. Mountain pine beetle (MPB) host suitability rating was provided by Ullah et al. [75]﻿ with the lowest rating being associated with trees least suitable for MPB colonization (i.e., trees more resistant to MPB) and the highest rating associated with trees most suitable for MPB attack (i.e., trees more susceptible to MPB). Both, WGR and MPB four-category ratings, were further transformed into a respective continuous normal score following Gianola and Norton [76].

The dendrochronological growth decline index (DECL) (Sebastian-Azcona et al. submitted) was calculated from tree ring information extracted from the same increment cores used for wood quality analysis. DECL describes the long-term growth reduction of trees that experienced multiple drought episodes by comparing their growth during the last 5 years of the study period to the growth during the 5-year period of maximum growth (1997–2001) prior to the first severe drought event that occurred in 2002. DECL was calculated from tree ring measurements as follows: *DECL* = *BAI*_*max*_/*BAI*_*last*_, where *BAI*_*max*_ is the average annual basal area increment (*BAI*) during 1997–2001 and *BAI*_*last*_ is the average *BAI* between 2012 and 2016. Trees with DECL values close to 1 represent non-declining trees that maintained a constant growth throughout their lifetime, while trees with larger DECL values were those who experienced a severe growth reduction compared to their early growth.

The two residual outside slabs of the increment core, retained after the pneumatic processing of the specimens for WD, were used to capture stable carbon isotope ratio (δ^13^C) variation across the lifespan of the tree. A detailed description of how these slabs were processed and analyzed can be found in Cappa et al. [7]. δ^13^C values were normalized and reported relative to the Vienna Pee Dee Belemnite.

Secondary chemical compounds (mainly monoterpenes) were identified and quantified from phloem tissue collected from each tree. Briefly, at each test site in July 2017, phloem samples from the main stem were taken at a height of 1 m using a 1.9 cm punch to the depth of the cambium, on the north-facing side of each tree. Samples were kept at − 40 °C and ground in liquid nitrogen to a powder for extraction. Hexane-extractable compounds were identified and quantified with a gas chromatograph-flame ionization detector following Klutsch et al. [77]. A total of 15 compounds were identified but only six monoterpenes (α-pinene, β-pinene, myrcene, limonene, β-phellandrene, and terpinolene) concentrations were employed in this study, including the sum of all hexane-extractable compound concentrations (total monoterpenes). These six monoterpenes along with the total concentration had the highest relative composition in trees and have known biological importance for defense against MPB [78]. The concentration of 3-carene was also in this list, but it did not fit model assumptions and was removed from all analyses.

Prior to model fitting, logarithmic transformation was used to DECL and all monoterpene compounds to improve data normality. With the exception of the univariate quantitative genomic analyses (i.e., single-trait and single-site models, see below Eq. [1]), all phenotypic data were spatially adjusted (e.g., [21, 79]) using the design effects. That is, following Cappa et al. [72], design adjusted phenotype data were obtained for each tree, trait and test site, by subtracting the estimated replication and set nested within replication effects from the original phenotype. Finally, data of all traits were standardized to have zero mean and unit variance. See Supplementary Table S5 for trait list, number of trees for each trait, and summary statistics for each phenotypic trait in their original scale (i.e., without design adjustment). From the final set of 1490 trees (see below), the number of trees selected in each trial was 370 in JUDY, 337 in VIRG, 391 in SWAN, and 392 in TIME, and by replication within trial varied from 56 to 101.

### Sample selection and genotyping-by-sequencing (GBS)

As described in Cappa et al. [72], 40 OP families (out of 224) were selected to represent the range of high, average and low height variation at age-30, and sampling approximately ten individual trees per OP family per site (*n* = 1600) (see Cappa et al. [72] for details). Additionally, 35 potential forward selected trees, previously identified based on height BVs, and from an additional 28 OP families were also included for sequencing, resulting in a total of 1635 trees being sequenced from a total of 68 OP families.

A total of 1554 (out of the original 1635 trees) DNA extracted samples passed the DNA quality control and were genotyped using the genotyping-by-sequencing (GBS) platform [80] as described by Cappa et al. [72]. A final set of 1490 trees and 25,099 (25 K) SNPs was retained based on SNP data set filtering for 30% missing data and a minor allele frequency ≥ 1%. Missing data were imputed using the mean allele frequency [57]. SNPs were generated using the reference-free UNEAK pipeline, due to the lack of a lodgepole pine genome reference assembly.

### Quantitative genomic analyses

In order to estimate the quantitative genetic parameters, including heritability and genetic correlations within- and across-sites, the following single-trait (ST) single-site individual-tree mixed model was fitted:

1 $$\boldsymbol{y}=\boldsymbol{X}\boldsymbol{\upbeta } +{\boldsymbol{Z}}_{\boldsymbol{d}}\boldsymbol{d}+{\boldsymbol{Z}}_{\boldsymbol{a}}\boldsymbol{a}+\boldsymbol{e}$$

where, ***y*** is the vector of phenotypic data, **β** is the vector of fixed effect of genetic groups to account for the means of the provenances; ***d*** is the vector of random design effects, replications and set nested within replication, given that in general, just one single tree was sampled from each 4-tree row plot, the plot effects were not fitted; ***a*** is the vector of random additive genetic effects following a normal distribution with zero mean and (co)variance matrix ${\boldsymbol{G}\upsigma}_{\boldsymbol{a}}^{\mathbf{2}}$, where ***G*** is the genomic relationship matrix (***G***-matrix, see below) based on 25 K SNPs and ${\upsigma}_{\boldsymbol{a}}^{\mathbf{2}}$ is the genetic variance; and ***e*** is the vector of the random residual effect following a normal distribution with zero mean and (co)variance matrix ${\upsigma}_{\boldsymbol{e}}^{\mathbf{2}}$, where ${\upsigma}_e^2$ is the residual error variance. ***X***, ***Z***_***d***_, and ***Z***_***a***_, are incidence matrices relating fixed and random effects to measurements in vector ***y***.

The genomic relationship matrix (***G***-matrix) based on 25 K SNPs was calculated as:

$$\boldsymbol{G}=\frac{{\mathbf{WW}}^{\prime }}{2\sum {\mathrm{p}}_{\mathrm{i}}\left(1-{\mathrm{p}}_{\mathrm{i}}\right)}$$

where, **W** is the *n* × *m* (*n* = number of individuals, *m* = number of SNPs) rescaled genotype matrix following **M** - **P**, where **M** is the genotype matrix containing genotypes coded as 0, 1, and 2 according to the number of alternative alleles, and **P** is a vector of twice the allelic frequency, p_i_.

Genetic correlations between traits measured from the same individual, and genetic correlations between sites, considering measurements from different sites as different traits, were estimated based on the following multiple-trait (MT) individual-tree mixed model:

2 $$\left[\begin{array}{c}{\boldsymbol{y}}_1^{\ast}\\ {}\vdots \\ {}{\boldsymbol{y}}_j^{\ast}\end{array}\right]=\left[\begin{array}{ccc}{\boldsymbol{X}}_1& \cdots & \mathbf{0}\\ {}\vdots & \ddots & \vdots \\ {}\mathbf{0}& \cdots & {\boldsymbol{X}}_j\end{array}\right]\left[\begin{array}{c}{\boldsymbol{\upbeta}}_1\\ {}\vdots \\ {}{\boldsymbol{\upbeta}}_j\end{array}\right]+\left[\begin{array}{ccc}{{\boldsymbol{Z}}_{\boldsymbol{a}}}_1& \cdots & \mathbf{0}\\ {}\vdots & \ddots & \vdots \\ {}\mathbf{0}& \cdots & {{\boldsymbol{Z}}_{\boldsymbol{a}}}_j\end{array}\right]\left[\begin{array}{c}{\boldsymbol{a}}_1\\ {}\vdots \\ {}{\boldsymbol{a}}_j\end{array}\right]+\left[\begin{array}{c}{\boldsymbol{e}}_1\\ {}\vdots \\ {}{\boldsymbol{e}}_j\end{array}\right]$$

where, $\left[{\boldsymbol{y}}_{1\kern0.5em }^{\ast \prime }|\mathbf{\cdots}|\kern0.5em {\boldsymbol{y}}_j^{\ast \prime}\right]$ is a *n* × 1 vector that included the spatially adjusted phenotypes of all the individual-tree measured on all traits (*j* = HT, DBH, WGR, WD, δ^13^C, MPB, α-pinene, β-pinene, myrcene, limonene, β-phellandrene, terpinolene, and total monoterpenes) or sites (*j* = JUDY, VIRG, SWAN, and TIME); the genetic group effects for all traits or sites are included in the vector $\left[{\boldsymbol{\upbeta}}_{1\kern0.5em }^{\prime }|\mathbf{\cdots}|\kern0.5em {\boldsymbol{\upbeta}}_j^{\prime}\right]$ of order *p* × 1; the additive genetic effects (breeding values) of all individuals (i.e., parents without records plus offspring with data in $\left[{\boldsymbol{y}}_{1\kern0.5em }^{\ast \prime }|\mathbf{\cdots}|\kern0.5em {\boldsymbol{y}}_j^{\ast \prime}\right]$) for all traits or sites are included in the vector $\left[{\boldsymbol{a}}_{1\kern0.5em }^{\prime }|\mathbf{\cdots}|\kern0.5em {\boldsymbol{a}}_j^{\prime}\right]$ of order *q* × 1, and $\left[{\boldsymbol{e}}_{1\kern0.5em }^{\prime }|\mathbf{\cdots}|\kern0.5em {\boldsymbol{e}}_j^{\prime}\right]$ is the residual vector of order *n* × 1. ***X***_**1**_ ***⊕ ⋯ ⊕ X***_***j***_ (of order *n* × *p*), and ${\boldsymbol{Z}}_{{\boldsymbol{a}}_{\mathbf{1}}}\oplus \boldsymbol{\cdots}\oplus {\boldsymbol{Z}}_{{\boldsymbol{a}}_{\boldsymbol{j}}}$ (of order *n* × *q*) are incidence matrices of zeros and ones relating observations of the *j*th trait or site in $\left[{\boldsymbol{y}}_{1\kern0.5em }^{\ast \prime }|\mathbf{\cdots}|\kern0.5em {\boldsymbol{y}}_j^{\ast \prime}\right]$ to elements of $\left[{\boldsymbol{\upbeta}}_{1\kern0.5em }^{\prime }|\mathbf{\cdots}|\kern0.5em {\boldsymbol{\upbeta}}_j^{\prime}\right]$ and $\left[{\boldsymbol{a}}_{1\kern0.5em }^{\prime }|\mathbf{\cdots}|\kern0.5em {\boldsymbol{a}}_j^{\prime}\right]$, respectively. The symbols ⨁ and ‘indicate the direct sum of matrices and transpose operation, respectively. Finally, the expected value and variance-covariance matrix of the additive genetic effects in model [2] are respectively equal to:

$$\boldsymbol{E}\left[\begin{array}{c}{\boldsymbol{a}}_i\\ {}\vdots \\ {}{\boldsymbol{a}}_j\end{array}\right]=\left[\begin{array}{c}\mathbf{0}\\ {}\vdots \\ {}\mathbf{0}\end{array}\right],\kern0.5em \boldsymbol{Var}\left[\begin{array}{c}{\boldsymbol{a}}_i\\ {}\vdots \\ {}{\boldsymbol{a}}_j\end{array}\right]=\left[\begin{array}{ccc}{\upsigma}_{{\boldsymbol{a}}_{{ii}}}^2\boldsymbol{G}& \cdots & {\upsigma}_{{\boldsymbol{a}}_{{ij}}}\boldsymbol{G}\\ {}\vdots & \ddots & \vdots \\ {}{\upsigma}_{{\boldsymbol{a}}_{{ji}}}\boldsymbol{G}& \cdots & {\upsigma}_{{\boldsymbol{a}}_{{jj}}}^2\boldsymbol{G}\end{array}\right]=\left[\begin{array}{ccc}{\upsigma}_{{\boldsymbol{a}}_{{ii}}}^2& \cdots & {\upsigma}_{{\boldsymbol{a}}_{{ij}}}\\ {}\vdots & \ddots & \vdots \\ {}{\upsigma}_{{\boldsymbol{a}}_{{ji}}}& \cdots & {\upsigma}_{{\boldsymbol{a}}_{{jj}}}^2\end{array}\right]\bigotimes \boldsymbol{G}$$

where, ${\upsigma}_{{\boldsymbol{a}}_{\boldsymbol{ii}}}^2$ and ${\upsigma}_{{\boldsymbol{a}}_{\boldsymbol{jj}}}^2$ are the genetic variances for traits or sites *i* and *j* respectively; and, ${\upsigma}_{{\boldsymbol{a}}_{\boldsymbol{ij}}}$ is the genetic covariance between traits or sites *i* and *j*. The symbol ***⨂*** indicates the Kronecker products of matrices. The expected value and variance-covariance matrix of ***e*** were equal to:

$$\boldsymbol{E}\left[\begin{array}{c}{\boldsymbol{e}}_i\\ {}\vdots \\ {}{\boldsymbol{e}}_j\end{array}\right]=\left[\begin{array}{c}\mathbf{0}\\ {}\vdots \\ {}\mathbf{0}\end{array}\right],\kern0.5em \boldsymbol{Var}\left[\begin{array}{c}{\boldsymbol{e}}_i\\ {}\vdots \\ {}{\boldsymbol{e}}_j\end{array}\right]=\left[\begin{array}{ccc}{\upsigma}_{{\boldsymbol{e}}_{{ii}}}^2\boldsymbol{I}& \cdots & {\upsigma}_{{\boldsymbol{e}}_{{ij}}}\boldsymbol{I}\\ {}\vdots & \ddots & \vdots \\ {}{\upsigma}_{{\boldsymbol{e}}_{{ji}}}\boldsymbol{I}& \cdots & {\upsigma}_{{\boldsymbol{e}}_{{jj}}}^2\boldsymbol{I}\end{array}\right]=\left[\begin{array}{ccc}{\upsigma}_{{\boldsymbol{e}}_{{ii}}}^2& \cdots & {\upsigma}_{{\boldsymbol{e}}_{{ij}}}\\ {}\vdots & \ddots & \vdots \\ {}{\upsigma}_{{\boldsymbol{e}}_{{ji}}}& \cdots & {\upsigma}_{{\boldsymbol{e}}_{{jj}}}^2\end{array}\right]\bigotimes \boldsymbol{I}$$

The residual variances for traits or sites *i* and *j* were ${\upsigma}_{{\boldsymbol{e}}_{{i}}}^2$, and ${\upsigma}_{{\boldsymbol{e}}_{{j}}}^2$, respectively, and ${\upsigma}_{{\boldsymbol{e}}_{{ij}}}$ was the residual covariance between traits *i* and *j*. Given that the sites were assessed separately, the residual covariances across-sites were assumed to be zero.

The individual-trait narrow-sense heritability (${\hat{h}}^2$) and genetic correlations (${\hat{r}}_a$) between traits, or sites *i* and *j*, were estimated as:

$${\hat{h}}^2=\frac{{\hat{\sigma}}_a^2}{{\hat{\sigma}}_a^2+{\hat{\sigma}}_e^2};\kern0.75em {\hat{r}}_a=\frac{{\hat{\sigma}}_{a_{i,j}}}{\sqrt{{\hat{\sigma}}_{a_{i,i}}^2\times {\hat{\sigma}}_{a_{j,j}}^2}}$$

where ${\hat{\upsigma}}_{\boldsymbol{a}}^2$ is the estimated of variances for the additive genetic effects, and ${\hat{\upsigma}}_{\boldsymbol{e}}^2$ is the estimated residual errors. Visualization of genetic correlations between traits was done using the corrplot function in R-package corrplot [81].

Univariate [1] and multivariate [2] models were fitted with the R-package (www.r-project.org) ‘breedR’ [82] using the function remlf90, which is based in the REMLF90 (for the Expectation-Maximization algorithm, EM) and AIREMLF90 (for the Average Information algorithm, AI) of the BLUPF90 family [83]. The EM algorithm was followed by one iteration with the AI algorithm to compute the approximate standard errors of variance components [84]. The program preGSf90, also from the BLUPF90 family [83], was used to calculate the inverse of the ***G***-matrix from the 25 K SNPs, and then used to fit models [1] and [2].

### GWA analyses

Single- and multiple-trait GWA analyses were performed using models [1] and [2], respectively, fitted in the breedR R-package. The only difference from these models was that the fixed effects of genetic groups and random design effects were not considered. Previous analyses using the heat map and principal component analysis of the genomic relationship matrix of the 1490 genotyped trees (not shown) revealed that differences attributable to provenance origins were negligible. For instance, the first principal component (PC) accounted for < 0.7% of the total variation. In addition, design random effects (i.e., replications and set nested within replication) were also not fitted in the GWA analyses, given that adjusted phenotypes were used. The two traits that showed inconsistent and imprecise genotype by environment interactions (G × E) were MFA and DECL and were therefore removed from further multiple-trait GWA and GP analyses (see Results). Thus, G × E was not fitted to simplify the multiple-trait models.

The *p*-value for each *k* SNP from each individual trait using the ST and MT models was computed with the formula [85]:

$$p\_{\mathrm{value}}_k=2\left(1-\Phi \left(\frac{{\hat{g}}_k}{sd\left({\hat{g}}_k\right)}\right)\right)$$

where $sd\left({\hat{g}}_k\right)$ is the standard deviation of the SNP effect estimate (${\hat{g}}_k$) ($sd\left({\hat{g}}_k\right)=\sqrt{Var\left({\hat{g}}_k\right)}$), $Var\left({\hat{g}}_k\right)$ is the variance of the estimated SNP effects, and *Ф*(.) is the cumulative density function of the normal distribution (see [85] for $Var\left({\hat{g}}_k\right)$ (expression 5) calculation details). The SNP effects for each trait and each ST and MT model were obtained from a linear transformation of the genomic breeding values in the vector ***a*** of model [1, 2] (expression 3 in [85]). Following Gualdrón Duarte [85], a customized R-script was written to obtain the ***p***-values from each ST and MT model. Positive associations were determined at the nominal *p*-value < 0.05 level, and Bonferroni correction was used to control false positive associations in the multiple comparison procedure. We, therefore, selected a -logP value of 5.7, derived by dividing the *p*-value = 0.05 by the total number of testing SNP markers in the analysis *N* = 25,099.

### Genomic prediction

Five statistical methods were assessed for their ability to predict the genomic breeding values using single-trait (ST) models for all traits studied. These methods included BayesC [86], Bayesian Lasso (BLasso, [87]), Bayesian ridge regression (BRR, [88]), genomic best linear unbiased prediction (GBLUP, [89]), and the non-parametric reproducing kernel Hilbert space (RKHS) regression (e.g., [90]). All ST models were run using the BGLR function of the BGLR R-package [91]. For ST-GP models, a single Gibbs chain of 20,000 iterations was sampled, the first 2000 iterations were discarded due to “burn-in”, and a thinning interval (thin) of 100 was used to compute posterior means.

As mentioned, based on the quantitative genomic results obtained from the multiple-site analyses using Eq. [2] for each trait (see Results), all further MT-GP analyses were carried out without considering MFA and DECL due to their inconsistent genotype by environment interactions. The MT-GP models were performed using two statistical methods that produced the best predictive performance in the previous ST-GP analysis (RKHS, see below) and represent the most commonly used GP approach in forest tree studies (GBLUP). The MT-RHKS and -GBLUP models were run using the Multitrait function of the BGLR R-package [91]. That is, RKHS regression models were fitted using a linear GBLUP kernel (GBLUP), given that the use of RKHS along with the ***G***-matrix (see above) is equivalent to the mixed linear model of GBLUP [92], and a non-linear Gaussian kernel (RKHS). The non-linear kernel matrix (***K***) is defined as: $\boldsymbol{K}\left({\boldsymbol{x}}_{{i}},{\boldsymbol{x}}_{{{i}}^{\acute{\mkern6mu}}}\right)={\boldsymbol{e}}^{-\left(\boldsymbol{h}\ast {\boldsymbol{d}}_{{{i}{i}}^{\acute{\mkern6mu}}}^{\mathbf{2}}\right)}$, where ${\boldsymbol{d}}_{{{ii}}^{\acute{\mkern6mu}}}^{\mathbf{2}}$ points out the squared Euclidean distance between individuals *i* and *i*^´^. The rate of decay imposed by the bandwidth parameter ***h*** was estimated using the ST model for each trait, where 11 out of the studied 15 traits showed the highest predictive ability to the ***h*** value equal to 0.5 (see [92]), for the two RKHS regression models fitted to study the MT-GP approaches details). For MT-GBLUP and RKHS GP models, a single Gibbs chain of 200,000 iterations was sampled, the first 1000 iterations were discarded due to “burn-in”, and a sample interval (thin) of 100 was used to compute posterior means.

In order to evaluate and compare the accuracy and bias of the genomic predictions of the studied traits, a 10-fold cross-validation analysis was conducted across all the ST and MT parametric and non-parametric GP models, where one subsample was used as the validation set, and the remaining nine samples as the training set. A total of 5 replicates were conducted at each fold. In the MT cross-validation analysis, when a trait is predicted for a tree in the validation population, the phenotypic measurements for the other traits are available for the tree in the validation set (trait-assisted GP, [26]).

Predictive ability was estimated by evaluating the Pearson correlation coefficient between the predicted breeding values of the validation trees and the adjusted phenotypes. Then, the prediction accuracy was calculated for each ST and MT model and trait as the predictive ability divided by the square root of the narrow-sense heritably of each trait, computed using the MT-GBLUP model [93]. The prediction bias was calculated by the regression coefficient between the observed adjusted phenotype and the predicted with each ST and MT-GP model. A regression coefficient equal to one is considered to have no bias, while a coefficient greater or smaller than one indicates deflated or inflated predictions. A customized R-script was written to automate the cross-validation analyses for each ST and MT-GP model.

An analysis of variance (ANOVA) using a linear model with fixed effects of method and replication, and Tukey’s multiple comparison tests were performed at a significance level α = 0.05, to test the significance of the difference in prediction accuracy and prediction bias between the different ST and MT models performed for each trait.

## Supplementary Information

**Additional file 1.**

## Data Availability

Genotyping-by-sequencing (GBS) raw reads used in this study have been deposited in NCBI SRA BioProject - PRJNA715165: https://www.ncbi.nlm.nih.gov/bioproject/715165. The pedigree and phenotypic data used and analysed during the current study are available in the GitHub repository: https://github.com/RESFOR/quantitative_genetics_R/blob/main/Lodgepole_Pine_Phenotypic_and_Pedigree_DATA.txt. Our field studies and experimental research on plants, including the collection of any plant material, complied with all institutional, national, or international guidelines and legislation. Voucher specimens were not taken for the thousands of field trees sampled and described in the manuscript. A unique identifier was used to label each tree in the field in order to maintain their genetic identity across genomic and phenotypic measurements. Tree identity is ultimately retained by the program owners and in the Government of Alberta archives.

## Abbreviations

AIC: Akaike information criterion
BVs: Breeding Values
DBH: Diameter at Breast Height
DECL: Decline index (DECL)
GBLUP: Genomic Best Linear Unbiased Prediction
GP: Genomic Prediction
GWA: Genome-Wide Association
G × E: Genotype by Environment interactions
HT: Tree Height
JUDY: Judy Creek
MFA: Microfibril Angle
MPB: Mountain Pine Beetle
MT: Multiple-Trait
RKHS: Reproducing Kernel Hilbert Spaces
RR-BLUP: Ridge Regression Best Linear Unbiased Predictor
ST: Single-Trait
SWAN: Swan Hills
TIME: Timeau
VIRG: Virginia Hills
WD: Wood Density
WGR: Western Gall Rust
δ^13^C: Stable carbon isotope ratio
