# Supplementary material for: Multiple-trait analyses improved the accuracy of genomic prediction and the power of genome-wide association of productivity and climate change-adaptive traits in lodgepole pine
Source: BMC Genomics. 2022 Jul 23;23:536. doi: 10.1186/s12864-022-08747-7 (PMC9308220; doi:10.1186/s12864-022-08747-7)
Supplement: Supplementary file 1 — Additional file 1. [file 12864_2022_8747_MOESM1_ESM.docx]

**Supplementary Information**

**Fig. S1** Manhattan plots for the single-trait ($●$ black) and multiple-trait ($●$ green) GWA analyses in the lodgepole pine population for the 13 traits studied. The vertical *y*-axis indicates -log_10_(*p*-value) and the horizontal *x*-axis indicates the single-SNPs. The red dash line are the Bonferroni correction *p*-value cutoff (---; Bonferroni correction, i.e., *p*-values < 1.99 × 10^-06^). See text for trait´s abbreviations.


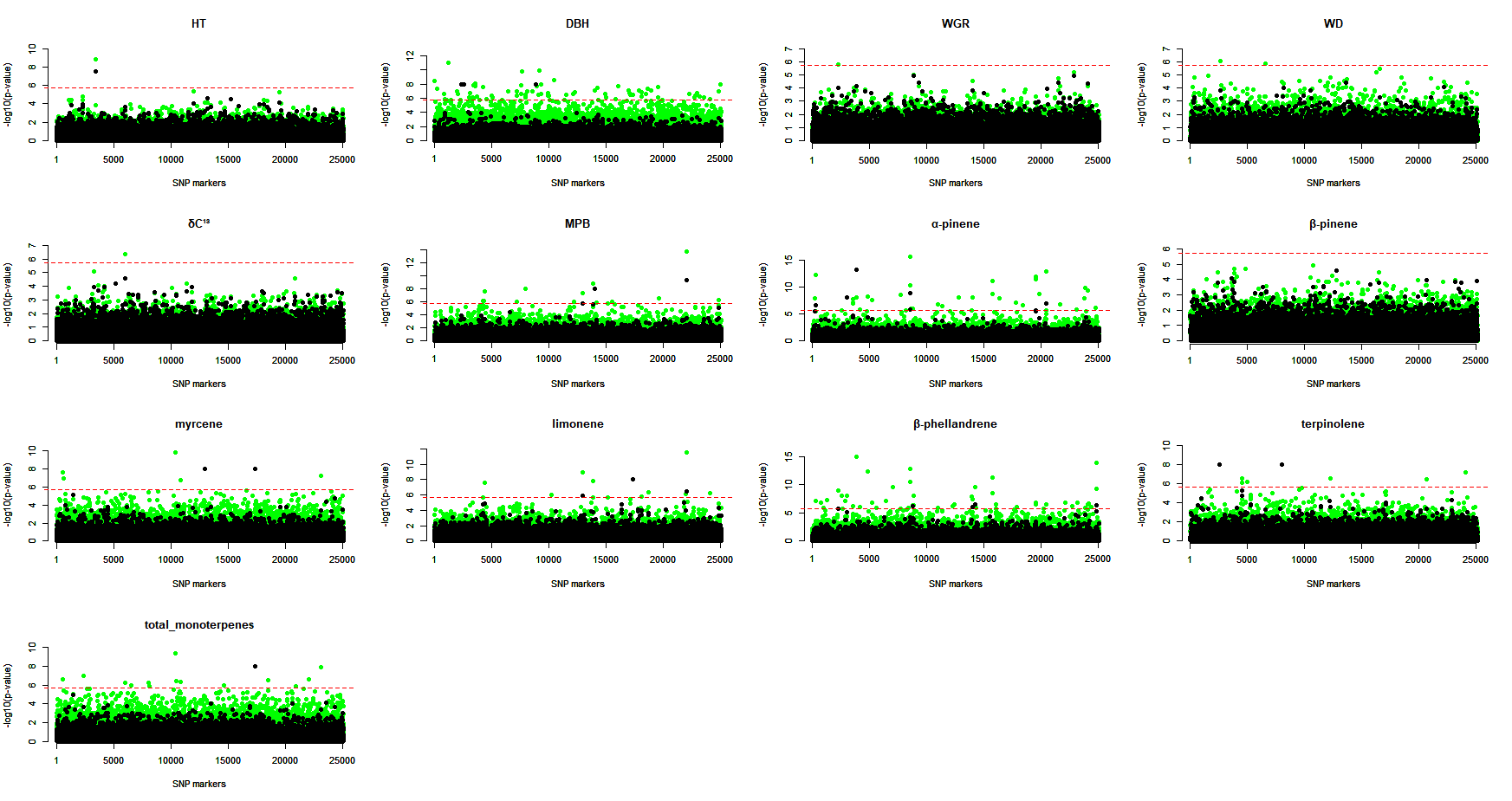


**Fig. S2** Location of the four lodgepole pine (black circles) progeny test sites in Alberta, Canada (see Table S1 for site abbreviations).


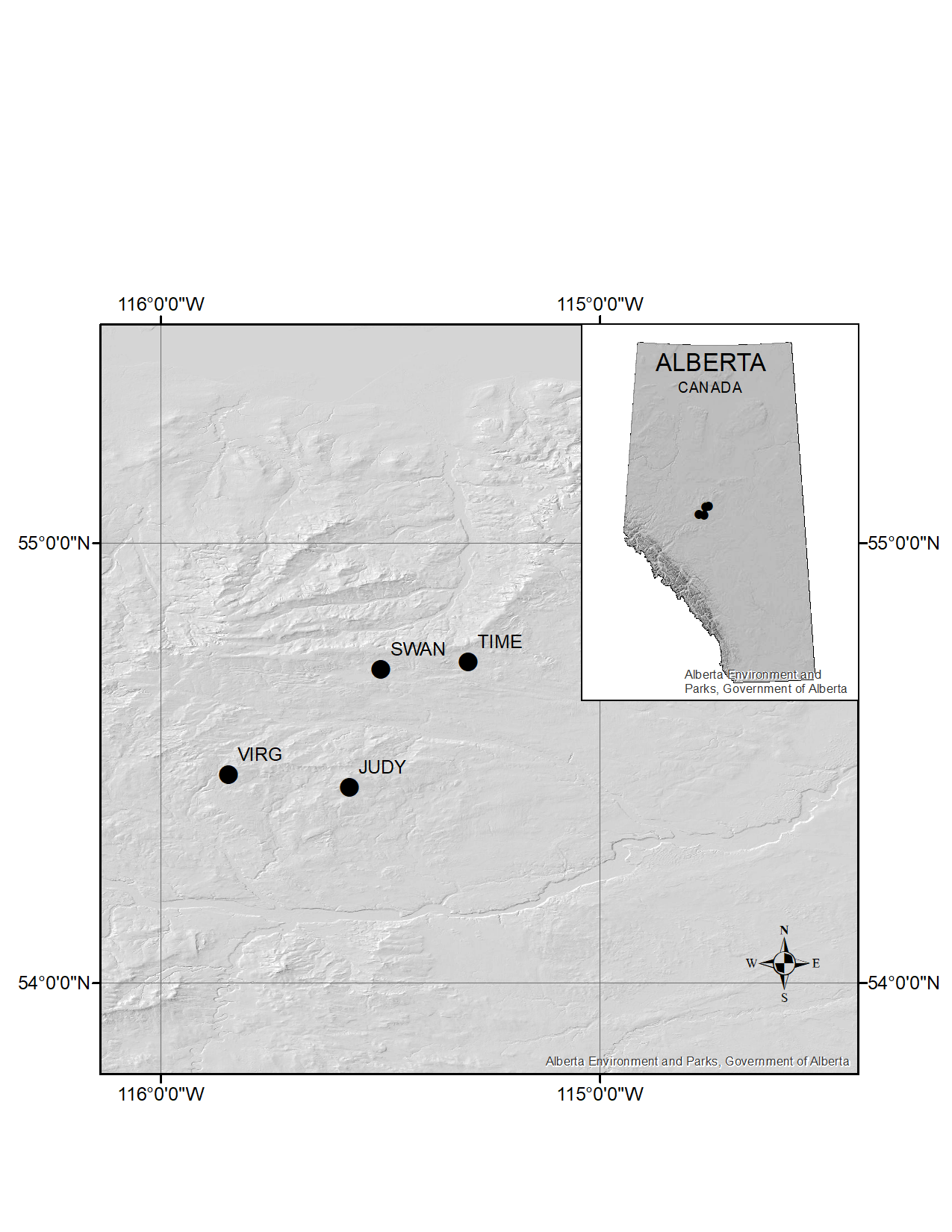


**Table S1** Genomic-based multiple-trait estimates of genetic correlation estimates (and their approximate standard errors) among the 15 traits studied. See text for site and trait abbreviations.

| **JUDY** | | | | | | | | | | | | | | |
| --- | --- | --- | --- | --- | --- | --- | --- | --- | --- | --- | --- | --- | --- | --- |
|  | **HT** | **DBH** | **WD** | **MFA** | **WGR** | **MPB** | **DECL** | **δ^13^C** | **α-pinene** | **β-pinene** | **myrcene** | **Limonene** | **β-phellandrene** | **terpinolene** |
| **DBH** | 0.59 (0.03) |  |  |  |  |  |  |  |  |  |  |  |  |  |
| **WD** | 0.13 (0.03) | -0.17 (0.03) |  |  |  |  |  |  |  |  |  |  |  |  |
| **MFA** | -0.21 (0.03) | 0.00 (0.02) | -0.52 (0.03) |  |  |  |  |  |  |  |  |  |  |  |
| **WGR** | -0.31 (0.03) | -0.13 (0.03) | -0.22 (0.05) | 0.17 (0.05) |  |  |  |  |  |  |  |  |  |  |
| **MPB** | 0.13 (0.02) | 0.44 (0.02) | -0.03 (0.02) | 0.05 (0.02) | -0.34 (0.02) |  |  |  |  |  |  |  |  |  |
| **DECL*^b^*** | 0.05 (0.03) | 0.05 (0.02) | -0.27 (0.03) | -0.28 (0.02) | 0.58 (0.03) | -0.57 (0.04) |  |  |  |  |  |  |  |  |
| **δ^13^C** | 0.28 (0.03) | 0.18 (0.02) | 0.34 (0.03) | -0.17 (0.02) | -0.14 (0.03) | 0.00 (0.05) | 0.14 (0.02) |  |  |  |  |  |  |  |
| **α-pinene*^b^*** | 0.15 (0.03) | 0.36 (0.03) | 0.08 (0.03) | 0.49 (0.03) | -0.01 (0.03) | -0.22 (0.02) | -0.21 (0.03) | 0.07 (0.03) |  |  |  |  |  |  |
| **β-pinene*^b^*** | 0.03 (0.02) | 0.28 (0.02) | -0.04 (0.02) | 0.12 (0.02) | 0.13 (0.02) | -0.34 (0.01) | 0.23 (0.02) | -0.05 (0.02) | 0.65 (0.02) |  |  |  |  |  |
| **myrcene*^b^*** | 0.10 (0.03) | -0.04 (0.02) | 0.21 (0.03) | 0.21 (0.02) | -0.25 (0.03) | -0.53 (0.02) | -0.10 (0.02) | -0.21 (0.02) | 0.65 (0.03) | 0.61 (0.02) |  |  |  |  |
| **limonene*^b^*** | -0.02 (0.03) | -0.19 (0.02) | 0.10 (0.03) | -0.18 (0.02) | 0.55 (0.03) | -0.85 (0.02) | 0.34 (0.03) | -0.11 (0.02) | 0.31 (0.03) | 0.37 (0.02) | 0.48 (0.03) |  |  |  |
| **β-phellandrene*^b^*** | -0.08 (0.03) | -0.44 (0.02) | 0.07 (0.03) | 0.13 (0.02) | -0.33 (0.03) | -0.38 (0.02) | -0.18 (0.02) | -0.23 (0.02) | 0.08 (0.03) | 0.36 (0.02) | 0.65 (0.03) | 0.23 (0.02) |  |  |
| **terpinolene*^b^*** | 0.10 (0.03) | -0.27 (0.02) | 0.62 (0.03) | -0.15 (0.02) | -0.32 (0.02) | -0.12 (0.01) | -0.22 (0.02) | -0.04 (0.02) | 0.23 (0.02) | 0.44 (0.02) | 0.54 (0.02) | 0.07 (0.02) | 0.65 (0.02) |  |
| **total monoterpene*^b^*** | 0.11 (0.03) | -0.06 (0.02) | 0.18 (0.02) | 0.25 (0.02) | -0.28 (0.02) | -0.55 (0.02) | -0.23 (0.02) | -0.06 (0.02) | 0.69 (0.03) | 0.68 (0.02) | 0.92 (0.03) | 0.46 (0.02) | 0.71 (0.02) | 0.58 (0.02) |
| **VIRG** | | | | | | | | | | | | | | |
| **DBH** | 0.63 (0.04) |  |  |  |  |  |  |  |  |  |  |  |  |  |
| **WD** | 0.50 (0.04) | 0.03 (0.05) |  |  |  |  |  |  |  |  |  |  |  |  |
| **MFA** | 0.25 (0.05) | 0.24 (0.05) | 0.21 (0.05) |  |  |  |  |  |  |  |  |  |  |  |
| **WGR** | 0.00 (0.06) | -0.35 (0.05) | 0.21 (0.05) | 0.19 (0.05) |  |  |  |  |  |  |  |  |  |  |
| **MPB** | -0.22 (0.05) | -0.20 (0.05) | -0.01 (0.05) | -0.19 (0.05) | -0.23 (0.05) |  |  |  |  |  |  |  |  |  |
| **DECL*^b^*** | -0.50 (0.04) | -0.35 (0.05) | -0.25 (0.05) | -0.55 (0.04) | -0.49 (0.04) | 0.26 (0.05) |  |  |  |  |  |  |  |  |
| **δ^13^C** | 0.08 (0.05) | 0.05 (0.05) | -0.12 (0.05) | 0.8 (0.02) | 0.17 (0.05) | -0.27 (0.05) | -0.34 (0.05) |  |  |  |  |  |  |  |
| **α-pinene*^b^*** | -0.03 (0.05) | -0.28 (0.05) | 0.34 (0.05) | 0.05 (0.05) | -0.25 (0.05) | 0.2 (0.05) | 0.57 (0.04) | 0.06 (0.05) |  |  |  |  |  |  |
| **β-pinene*^b^*** | -0.25 (0.05) | -0.19 (0.05) | 0.19 (0.05) | 0.08 (0.05) | -0.25 (0.05) | -0.32 (0.05) | 0.46 (0.04) | 0.15 (0.05) | 0.69 (0.03) |  |  |  |  |  |
| **myrcene*^b^*** | -0.19 (0.05) | -0.15 (0.05) | 0.05 (0.06) | 0.04 (0.05) | -0.11 (0.05) | -0.22 (0.05) | 0.41 (0.05) | 0.14 (0.05) | 0.30 (0.05) | 0.72 (0.03) |  |  |  |  |
| **limonene*^b^*** | 0.02 (0.05) | -0.13 (0.05) | 0.07 (0.05) | 0.16 (0.05) | 0.53 (0.04) | -0.77 (0.02) | -0.15 (0.05) | 0.17 (0.05) | 0.07 (0.05) | 0.51 (0.04) | 0.50 (0.04) |  |  |  |
| **β-phellandrene*^b^*** | -0.23 (0.05) | 0.02 (0.05) | -0.17 (0.05) | -0.07 (0.05) | -0.05 (0.06) | -0.49 (0.04) | 0.14 (0.05) | 0.17 (0.05) | -0.14 (0.05) | 0.52 (0.04) | 0.79 (0.02) | 0.59 (0.04) |  |  |
| **terpinolene*^b^*** | 0.01 (0.05) | 0.00 (0.05) | 0.54 (0.04) | 0.20 (0.05) | -0.09 (0.05) | 0.22 (0.05) | 0.12 (0.05) | 0.12 (0.05) | 0.23 (0.05) | 0.34 (0.05) | 0.56 (0.04) | 0.00 (0.06) | 0.40 (0.05) |  |
| **Total monoterpene*^b^*** | -0.24 (0.05) | -0.29 (0.05) | 0.19 (0.05) | 0.00 (0.05) | -0.11 (0.05) | -0.35 (0.05) | 0.35 (0.05) | 0.20 (0.05) | 0.43 (0.05) | 0.84 (0.02) | 0.87 (0.01) | 0.56 (0.04) | 0.79 (0.02) | 0.58 (0.04) |
| **SWAN** | | | | | | | | | | | | | | |
| **DBH** | 0.51 (0.04) |  |  |  |  |  |  |  |  |  |  |  |  |  |
| **WD** | 0.40 (0.04) | 0.05 (0.05) |  |  |  |  |  |  |  |  |  |  |  |  |
| **MFA** | 0.07 (0.05) | 0.04 (0.05) | 0.19 (0.05) |  |  |  |  |  |  |  |  |  |  |  |
| **WGR** | -0.07 (0.05) | -0.24 (0.05) | -0.3 (0.04) | -0.19 (0.05) |  |  |  |  |  |  |  |  |  |  |
| **MPB** | -0.02 (0.05) | 0.58 (0.03) | -0.16 (0.05) | 0.19 (0.05) | 0.31 (0.05) |  |  |  |  |  |  |  |  |  |
| **DECL*^b^*** | -0.42 (0.04) | -0.07 (0.05) | 0.00 (0.05) | -0.50 (0.04) | 0.33 (0.05) | -0.22 (0.05) |  |  |  |  |  |  |  |  |
| **δ^13^C** | 0.42 (0.04) | 0.39 (0.04) | 0.13 (0.05) | 0.20 (0.05) | -0.09 (0.05) | -0.03 (0.05) | 0.24 (0.05) |  |  |  |  |  |  |  |
| **α-pinene*^b^*** | 0.30 (0.05) | 0.03 (0.05) | 0.48 (0.04) | -0.29 (0.05) | -0.12 (0.05) | -0.39 (0.04) | 0.02 (0.05) | 0.22 (0.05) |  |  |  |  |  |  |
| **β-pinene*^b^*** | 0.08 (0.05) | -0.32 (0.05) | 0.25 (0.05) | -0.02 (0.05) | -0.29 (0.05) | -0.62 (0.03) | 0.02 (0.05) | 0.19 (0.05) | 0.45 (0.04) |  |  |  |  |  |
| **myrcene*^b^*** | 0.08 (0.05) | -0.11 (0.05) | 0.03 (0.05) | 0.12 (0.05) | -0.49 (0.04) | -0.50 (0.04) | -0.06 (0.05) | 0.41 (0.04) | 0.47 (0.04) | 0.65 (0.03) |  |  |  |  |
| **limonene*^b^*** | -0.20 (0.05) | -0.34 (0.04) | 0.01 (0.05) | 0.02 (0.05) | -0.17 (0.05) | -0.58 (0.03) | 0.24 (0.05) | 0.37 (0.04) | 0.18 (0.05) | 0.49 (0.04) | 0.30 (0.05) |  |  |  |
| **β-phellandrene*^b^*** | -0.04 (0.05) | -0.20 (0.05) | -0.19 (0.05) | 0.07 (0.05) | -0.20 (0.05) | -0.29 (0.05) | 0.18 (0.05) | 0.19 (0.05) | -0.48 (0.04) | 0.24 (0.05) | 0.35 (0.04) | 0.07 (0.05) |  |  |
| **terpinolene*^b^*** | 0.18 (0.05) | 0.30 (0.05) | 0.08 (0.05) | -0.02 (0.05) | -0.26 (0.05) | 0.00 (0.05) | -0.07 (0.05) | -0.10 (0.05) | 0.06 (0.05) | 0.12 (0.05) | 0.26 (0.05) | -0.31 (0.05) | 0.16 (0.05) |  |
| **total monoterpene*^b^*** | 0.01 (0.05) | -0.23 (0.05) | 0.18 (0.05) | 0.11 (0.05) | -0.50 (0.04) | -0.64 (0.03) | 0.08 (0.05) | 0.36 (0.04) | 0.41 (0.04) | 0.81 (0.02) | 0.92 (0.01) | 0.45 (0.04) | 0.45 (0.04) | 0.31 (0.05) |
| **TIME** | | | | | | | | | | | | | | |
| **DBH** | 0.60 (0.03) |  |  |  |  |  |  |  |  |  |  |  |  |  |
| **WD** | 0.60 (0.04) | 0.09 (0.05) |  |  |  |  |  |  |  |  |  |  |  |  |
| **MFA** | 0.26 (0.05) | 0.38 (0.04) | -0.12 (0.05) |  |  |  |  |  |  |  |  |  |  |  |
| **WGR** | -0.13 (0.05) | -0.16 (0.05) | 0.00 (0.05) | -0.19 (0.05) |  |  |  |  |  |  |  |  |  |  |
| **MPB** | 0.00 (0.05) | 0.32 (0.05) | 0.10 (0.05) | 0.28 (0.05) | 0.18 (0.05) |  |  |  |  |  |  |  |  |  |
| **DECL*^b^*** | 0.03 (0.05) | -0.26 (0.05) | 0.25 (0.05) | -0.19 (0.05) | 0.39 (0.04) | -0.15 (0.05) |  |  |  |  |  |  |  |  |
| **δ^13^C** | 0.55 (0.04) | 0.38 (0.04) | 0.21 (0.05) | 0.27 (0.05) | 0.41 (0.04) | 0.11 (0.05) | 0.33 (0.05) |  |  |  |  |  |  |  |
| **α-pinene*^b^*** | 0.28 (0.05) | 0.59 (0.03) | -0.12 (0.05) | -0.05 (0.05) | -0.18 (0.05) | 0.37 (0.04) | 0.11 (0.05) | 0.15 (0.05) |  |  |  |  |  |  |
| **β-pinene*^b^*** | 0.07 (0.05) | -0.13 (0.05) | 0.02 (0.05) | 0.12 (0.05) | -0.42 (0.04) | 0.14 (0.05) | 0.37 (0.04) | 0.08 (0.05) | 0.30 (0.05) |  |  |  |  |  |
| **myrcene*^b^*** | 0.33 (0.04) | 0.37 (0.04) | 0.25 (0.05) | -0.03 (0.05) | -0.36 (0.04) | -0.02 (0.05) | -0.07 (0.05) | 0.13 (0.05) | 0.19 (0.05) | 0.30 (0.05) |  |  |  |  |
| **limonene*^b^*** | 0.24 (0.05) | -0.03 (0.05) | 0.01 (0.05) | -0.07 (0.05) | -0.13 (0.05) | -0.83 (0.02) | 0.18 (0.05) | -0.08 (0.05) | -0.03 (0.05) | -0.08 (0.05) | -0.15 (0.05) |  |  |  |
| **β-phellandrene*^b^*** | 0.26 (0.05) | -0.33 (0.05) | 0.35 (0.04) | -0.22 (0.05) | -0.09 (0.05) | -0.22 (0.05) | -0.04 (0.05) | 0.21 (0.05) | -0.45 (0.04) | 0.17 (0.05) | 0.43 (0.04) | -0.19 (0.05) |  |  |
| **terpinolene*^b^*** | 0.03 (0.05) | 0.20 (0.05) | 0.49 (0.04) | -0.45 (0.04) | -0.14 (0.05) | 0.22 (0.05) | 0.04 (0.05) | -0.04 (0.05) | -0.02 (0.05) | -0.18 (0.05) | 0.54 (0.04) | -0.37 (0.04) | 0.20 (0.05) |  |
| **total monoterpene*^b^*** | 0.32 (0.05) | 0.25 (0.05) | 0.32 (0.05) | -0.09 (0.05) | -0.28 (0.05) | 0.24 (0.05) | 0.00 (0.05) | 0.18 (0.05) | 0.35 (0.05) | 0.46 (0.04) | 0.87 (0.01) | -0.35 (0.05) | 0.46 (0.04) | 0.43 (0.04) |

**Table S2** Genomic-based multiple-trait estimates of genetic correlation (and approximate standard errors) for each trait at each site for lodgepole pine. See text for site and trait abbreviations.

| **Trait** | **Site** | **JUDY** | **VIRG** | **SWAN** |
| --- | --- | --- | --- | --- |
| **HT** | **VIRG** | 0.69 (0.19) |  |  |
|  | **SWAN** | 0.77 (0.12) | 0.82 (0.13) |  |
|  | **TIME** | 0.66 (0.17) | 0.74 (0.18) | 0.97 (0.03) |
| **DBH** | **VIRG** | 0.49 (0.26) |  |  |
|  | **SWAN** | 0.92 (0.12) | 0.33 (0.31) |  |
|  | **TIME** | 0.89 (0.16) | 0.29 (0.36) | 0.95 (0.18) |
| **WGR** | **VIRG** | 0.98 (0.04) |  |  |
|  | **SWAN** | 0.99 (0.03) | 0.99 (0.02) |  |
|  | **TIME** | 0.98 (0.05) | 0.99 (0.03) | 0.99 (0.01) |
| **WD** | **VIRG** | 0.85 (0.16) |  |  |
|  | **SWAN** | 0.88 (0.14) | 0.52 (0.41) |  |
|  | **TIME** | 0.52 (0.22) | 0.59 (0.31) | 0.42 (0.33) |
| **MFA** | **VIRG** | -0.15 (0.48) |  |  |
|  | **SWAN** | -0.26 (0.43) | 0.88 (0.35) |  |
|  | **TIME** | -0.20 (0.93) | 0.76 (0.44) | 0.46 (0.57) |
| **δ^13^C** | **VIRG** | 0.91 (0.06) |  |  |
|  | **SWAN** | 0.73 (0.15) | 0.88 (0.08) |  |
|  | **TIME** | 0.86 (0.08) | 0.95 (0.04) | 0.95 (0.04) |
| **DECL*^b^*** | **VIRG** | -0.29 (1.50) |  |  |
|  | **SWAN** | 0.03 (1.15) | -0.52 (1.22) |  |
|  | **TIME** | -0.68 (0.46) | -0.34 (0.81) | -0.11 (0.96) |
| **MPB** | **VIRG** | 0.23 (0.46) |  |  |
|  | **SWAN** | 0.70 (0.37) | 0.78 (0.15) |  |
|  | **TIME** | 0.91 (0.43) | 0.51 (0.30) | 0.89 (0.15) |
| **α-pinene*^b^*** | **VIRG** | 0.86 (0.15) |  |  |
|  | **SWAN** | 0.97 (0.03) | 0.94 (0.08) |  |
|  | **TIME** | 0.73 (0.23) | 0.73 (0.25) | 0.77 (0.2) |
| **β-pinene*^b^*** | **VIRG** | ***a*** |  |  |
|  | **SWAN** | ***a*** | ***a*** |  |
|  | **TIME** | ***a*** | ***a*** | ***a*** |
| **myrcene*^b^*** | **VIRG** | -0.03 (0.49) |  |  |
|  | **SWAN** | 0.72 (0.21) | 0.63 (0.28) |  |
|  | **TIME** | 0.38 (0.54) | 0.53 (0.54) | 0.75 (0.41) |
| **limonene*^b^*** | **VIRG** | 0.26 (0.26) |  |  |
|  | **SWAN** | 0.91 (0.06) | 0.61 (0.17) |  |
|  | **TIME** | 0.91 (0.08) | 0.58 (0.20) | 0.98 (0.03) |
|  | **VIRG** | 0.81 (0.09) |  |  |
| **β-phellandrene*^b^*** | **SWAN** | 0.71 (0.12) | 0.92 (0.04) |  |
|  | **TIME** | 0.93 (0.04) | 0.88 (0.06) | 0.75 (0.1) |
| **terpinolene*^b^*** | **VIRG** | 0.69 (0.23) |  |  |
|  | **SWAN** | 0.81 (0.18) | 0.95 (0.04) |  |
|  | **TIME** | 0.50 (0.31) | 0.92 (0.06) | 0.86 (0.09) |
| **total_monoterpene*^b^*** | **VIRG** | 0.36 (0.55) |  |  |
|  | **SWAN** | 0.81 (0.25) | 0.82 (0.26) |  |
|  | **TIME** | 0.66 (0.43) | 0.84 (0.31) | 0.93 (0.2) |

***^a^*** Correlations and their approximate standard errors were not estimated due to lack of convergence.

***^b^*** Transformed data were used.

**Table S3** Average prediction accuracy (and approximate standard errors), and prediction bias using different genomic selection models for five single- and two multiple-trait models and each of the 13 traits studied in lodgepole pine. The maximum prediction accuracy values and prediction bias closest to 1 for the single- and multi-trait models are shown in bold. Total average followed by the same letter are not significantly different (α = 0.05) according to the Tukey test. See text for trait abbreviations.

|  | **Single-trait model** | | | | | **Multiple-trait model** | |
| --- | --- | --- | --- | --- | --- | --- | --- |
| **Trait/method** | **BayesC** | **BLasso** | **BRR** | **GBLUP** | **RKHS** | **GBLUP** | **RKHS** |
|  | **Prediction accuracy** | | | | | | |
| **HT** | 0.458 (0.011) | 0.461 (0.011) | 0.466 (0.011) | 0.462 (0.011) | **0.468 (0.011)** | 0.645 (0.010) | **0.657 (0.010)** |
| **DBH** | 0.313 (0.020) | 0.322 (0.018) | **0.327 (0.019)** | 0.323 (0.018) | 0.309 (0.019) | **0.590 (0.016**) | 0.577 (0.015) |
| **WGR** | 0.446 (0.014) | 0.445 (0.013) | 0.449 (0.013) | 0.447 (0.012) | **0.461 (0.014)** | **0.419 (0.015**) | 0.410 (0.013) |
| **WD** | 0.356 (0.017) | **0.365 (0.017)** | 0.364 (0.018) | 0.357 (0.017) | 0.360 (0.017) | **0.425 (0.016)** | 0.401 (0.017) |
| **δ^13^C** | 0.483 (0.014) | 0.493 (0.013) | 0.489 (0.014) | 0.487 (0.014) | **0.510 (0.015)** | **0.502 (0.013)** | 0.465 (0.012) |
| **MPB** | 0.336 (0.019) | 0.349 (0.020) | 0.347 (0.020) | 0.345 (0.019) | **0.384 (0.018)** | 0.683 (0.021) | **0.758 (0.020)** |
| **α-pinene** | 0.470 (0.016) | 0.483 (0.014) | 0.464 (0.015) | 0.465 (0.015) | **0.497 (0.015)** | 0.785 (0.013) | **0.935 (0.010)** |
| **β-pinene** | 0.462 (0.016) | 0.467 (0.015) | 0.466 (0.016) | 0.469 (0.016) | **0.516 (0.015)** | 0.706 (0.012) | **0.904 (0.010)** |
| **myrcene** | 0.326 (0.022) | 0.332 (0.023) | 0.335 (0.024) | 0.335 (0.023) | **0.368 (0.024)** | 0.781 (0.017) | **0.919 (0.014)** |
| **limonene** | 0.381 (0.015) | 0.385 (0.015) | 0.382 (0.015) | 0.384 (0.014) | **0.415 (0.015)** | 0.606 (0.016) | **0.724 (0.015)** |
| **β-phellandrene** | 0.483 (0.016) | 0.490 (0.015) | 0.486 (0.016) | 0.487 (0.015) | **0.500 (0.015)** | 0.746 (0.009) | **0.808 (0.008)** |
| **terpinolene** | 0.442 (0.018) | 0.445 (0.018) | 0.444 (0.018) | 0.445 (0.018) | **0.453 (0.020)** | **0.594 (0.016)** | 0.531 (0.016) |
| **total_monoterpenes** | 0.367 (0.024) | 0.371 (0.026) | 0.375 (0.027) | 0.376 (0.026) | **0.414 (0.027)** | 0.887 (0.018) | **1.044 (0.019)** |
| ***Total average*** | *0.410 (0.017)^a^* | *0.416 (0.017)^a^* | *0.415 (0.017)^a^* | *0.414 (0.017)^a^* | ***0.435 (0.017)****^a^* | *0.644 (0.015)^b^* | ***0.703 (0.014)****^c^* |
|  | **Prediction bias** | | | | | | |
| **HT** | 0.912 (0.029) | 0.915 (0.029) | 0.917 (0.029) | 0.903 (0.029) | **0.974 (0.028)** | 0.959 (0.015) | **0.973 (0.016)** |
| **DBH** | 0.678 (0.042) | 0.682 (0.043) | 0.689 (0.041) | 0.683 (0.041) | **0.695 (0.043)** | 0.952 (0.016) | **0.958 (0.016)** |
| **WGR** | 0.890 (0.033) | 0.895 (0.032) | 0.893 (0.034) | 0.878 (0.032) | **0.951 (0.034)** | 0.835 (0.032) | **0.865 (0.031)** |
| **WD** | 0.746 (0.049) | 0.764 (0.054) | 0.756 (0.052) | 0.727 (0.050) | **0.773 (0.052)** | 0.856 (0.025) | **0.865 (0.025)** |
| **δ^13^C** | 0.889 (0.029) | 0.911 (0.031) | 0.889 (0.03) | 0.884 (0.030) | **0.952 (0.031)** | 0.840 (0.024) | **0.864 (0.023)** |
| **MPB_rank** | 0.652 (0.057) | 0.677 (0.062) | 0.670 (0.059) | 0.663 (0.057) | **0.729 (0.057)** | 0.966 (0.014) | **0.972 (0.013)** |
| **α-pinene** | 0.865 (0.031) | 0.884 (0.030) | 0.858 (0.031) | 0.855 (0.031) | **0.925 (0.032)** | 0.992 (0.009) | **0.999 (0.009)** |
| **β-pinene** | 0.865 (0.032) | 0.875 (0.031) | 0.865 (0.033) | 0.869 (0.033) | **0.957 (0.032)** | 0.986 (0.010) | **0.995 (0.010)** |
| **myrcene** | 0.630 (0.054) | 0.614 (0.052) | 0.616 (0.052) | 0.617 (0.052) | **0.685 (0.052)** | 0.997 (0.004) | **0.999 (0.004)** |
| **limonene** | 0.823 (0.037) | 0.836 (0.036) | 0.819 (0.036) | 0.819 (0.035) | **0.883 (0.035)** | 0.982 (0.008) | **0.993 (0.008)** |
| **β-phellandrene** | 0.894 (0.038) | 0.903 (0.037) | 0.891 (0.040) | 0.890 (0.037) | **0.965 (0.040)** | 0.986 (0.010) | **1.000 (0.010)** |
| **terpinolene** | 0.812 (0.044) | 0.830 (0.045) | 0.813 (0.044) | 0.809 (0.044) | **0.849 (0.048)** | 0.977 (0.012) | **0.985 (0.013)** |
| **total_monoterpenes** | 0.673 (0.056) | 0.657 (0.054) | 0.660 (0.055) | 0.662 (0.055) | **0.726 (0.054)** | 0.993 (0.004) | **0.994 (0.004)** |
| ***Total average*** | *0.794 (0.041) ^a^* | *0.803 (0.041) ^a^* | *0.795 (0.041) ^a^* | *0.789 (0.041) ^a^* | ***0.851 (0.041)^b^*** | *0.948 (0.014) ^c^* | ***0.959 (0.014) ^c^*** |

**Table S4** Trial information including location, sites characteristics, climate, date of planting, and experimental design for the four lodgepole pine progeny test sites in Alberta, Canada.

| **Trial** | **JUDY** | **VIRG** | **SWAN** | **TIME** |
| --- | --- | --- | --- | --- |
| **Location** | Judy Creek | Virginia Hills | Swan Hills | Timeau |
| **Latitude (°N)** | 54°45’ | 54°47' | 54°71' | 54°73' |
| **Longitude (°W)** | 115° 57’ | 115°85' | 115°50' | 115°30' |
| **Elevation (m)** | 1110 | 1118 | 1036 | 1097 |
| **Soil texture** | Clay loam | Silty loam | Fine silty loam | Sandy loam |
| **MAT (°C)** | 2.7 | 2.6 | 2.3 | 2.6 |
| **MWMT (°C)** | 14.9 | 14.6 | 14.4 | 14.9 |
| **MAP (mm)** | 550 | 577 | 610 | 549 |
| **MSP (mm)** | 376 | 400 | 433 | 380 |
| **CMI (mm)** | 16 | 20 | 24 | 14 |
| **Planting date** | 03-08/06/1982 | 31/05-02/06/1982 | 8-11/06/1982 | 14-18/06/1982 |
| **Number of replicates** | 5 | 5 | 5 | 5 |
| **Number of sets** | 21 | 21 | 21 | 21 |
| **Number of rows** | 48 | 48 | 52 | 52 |
| **Number of columns** | 120 | 108 | 120 | 108 |
| **Initial number of trees** | 4655 | 5032 | 5016 | 5040 |
| **Survival at 30 years (%)** | 58 | 42 | 87 | 83 |

**Table S5** Phenotypic mean for the 15 phenotypic traits studied. Number of trees used for each trait used in the quantitative parameters analyses (***n***), and statistics: mean, median, standard deviation (SD), phenotypic coefficient of variation (CV), minimum (Min.), and maximum (Max.) values. See text for trait abbreviations.

| **Trait** | **Unit** | ***n*** | **Mean** | **SD** | **CV** | **Min.** | **Max.** |
| --- | --- | --- | --- | --- | --- | --- | --- |
| **HT** | cm | 1,490 | 1088.00 | 118.68 | 0.11 | 340 | 1480 |
| **DBH** | cm | 1,490 | 16.86 | 2.62 | 0.16 | 7.2 | 25.3 |
| **WGR** | Scale 1-4 | 1,490 | 1.9 | 1.11 | 0.58 | 1 | 4 |
| **WD** | kg.m^-3^ | 1,408 | 400.82 | 29.45 | 0.07 | 309.52 | 556.21 |
| **MFA** | ° | 1,489 | 33.54 | 4.01 | 0.12 | 29.53 | 59.7 |
| **δ^13^C** | - | 1,480 | -25.93 | 0.64 | -0.02 | -27.81 | -24.03 |
| **DECL** | - | 1,273 | 1.8 | 0.9 | 0.50 | 0.38 | 11.1 |
| **MPB** | Scale 1-4 | 1,475 | 2.98 | 1.08 | 0.36 | 1 | 4 |
| **α-Pinene** | ng mg^-1^ | 1,475 | 1887.31 | 2300 .08 | 1.22 | 74.7 | 38412.97 |
| **β-Pinene** | ng mg^-1^ | 1,475 | 1530.91 | 1802.59 | 1.18 | 55.5 | 17869.4 |
| **Myrcene** | ng mg^-1^ | 1,475 | 278.21 | 189.12 | 0.68 | 7.88 | 1586.58 |
| **Limonene** | ng mg^-1^ | 1,475 | 831.24 | 1385.61 | 1.67 | 21.57 | 14605.27 |
| **β-Phellandrene** | ng mg^-1^ | 1,475 | 8673.59 | 6359.89 | 0.73 | 55.89 | 52175.31 |
| **Terpinolene** | ng mg^-1^ | 1,471 | 269.86 | 281.15 | 1.04 | 7.81 | 2823.24 |
| **Total monoterpenes** | ng mg^-1^ | 1,475 | 16334.43 | 10105.81 | 0.62 | 543.37 | 76122.93 |
